# The Phytochemistry of Cherokee Aromatic Medicinal Plants

**DOI:** 10.3390/medicines5040121

**Published:** 2018-11-12

**Authors:** William N. Setzer

**Affiliations:** 1 Department of Chemistry, University of Alabama in Huntsville, Huntsville, AL 35899, USA; wsetzer@chemistry.uah.edu; Tel.: +1-256-824-6519; 2 Aromatic Plant Research Center, 230 N 1200 E, Suite 102, Lehi, UT 84043, USA

**Keywords:** Cherokee, Native American, traditional herbal medicine, chemical constituents, pharmacology

## Abstract

**Background:** Native Americans have had a rich ethnobotanical heritage for treating diseases, ailments, and injuries. Cherokee traditional medicine has provided numerous aromatic and medicinal plants that not only were used by the Cherokee people, but were also adopted for use by European settlers in North America. **Methods:** The aim of this review was to examine the Cherokee ethnobotanical literature and the published phytochemical investigations on Cherokee medicinal plants and to correlate phytochemical constituents with traditional uses and biological activities. **Results:** Several Cherokee medicinal plants are still in use today as herbal medicines, including, for example, yarrow (*Achillea millefolium*), black cohosh (*Cimicifuga racemosa*), American ginseng (*Panax quinquefolius*), and blue skullcap (*Scutellaria lateriflora*). This review presents a summary of the traditional uses, phytochemical constituents, and biological activities of Cherokee aromatic and medicinal plants. **Conclusions:** The list is not complete, however, as there is still much work needed in phytochemical investigation and pharmacological evaluation of many traditional herbal medicines.

## 1. Introduction

Natural products have been an important source of medicinal agents throughout history and modern medicine continues to rely on traditional knowledge for treatment of human maladies [1]. Traditional medicines such as Traditional Chinese Medicine [2], Ayurvedic [3], and medicinal plants from Latin America [4] have proven to be rich resources of biologically active compounds and potential new drugs. Several plant-derived drugs are in use today, including, for example, vinblastine (from *Catharanthus roseus* (L.) G. Don, used to treat childhood leukemia); paclitaxel (from *Taxus brevifolia* Nutt., used to treat ovarian cancer); morphine (from *Papaver somniferum* L., used to treat pain); and quinine (from *Cinchona* spp., used to treat malaria) [5]. Not only are phytochemicals useful medicines in their own right, but compounds derived from them or inspired by them have become useful medicines [6,7]. For example, *Artemisia annua* L., a plant originally used in Traditional Chinese Medicine to treat fever, is the source of artemisinin, a clinically-useful antimalarial sesquiterpenoid [8]; the antihypertensive drug reserpine, isolated from the roots of *Rauvolfia serpentina* (L.) Benth. ex Kurz., has been used in Ayurveda to treat insanity, epilepsy, insomnia, hysteria, eclampsia, as well as hypertension [9]; *Dysphania ambrosioides* (L.) Mosyakin and Clemants (syn. *Chenopodium ambrosioides* L.) is used in several Latin American cultures as an internal anthelmintic and external antiparasitic [4] and has shown promise for treatment of cutaneous leishmaniasis [10]. The biological activity of *D. ambrosioides* has been attributed to the monoterpenoid endoperoxide ascaridole.

Unfortunately, much of the traditional medicine knowledge of Native North American peoples has been lost due to population decimation and displacement from their native lands by European conquerors (see, for example: [11,12,13,14]). Nevertheless, there are still some remaining sources of information about Native American ethnobotany [15,16]. In addition, there are several sources of Cherokee ethnobotany [17,18,19,20,21,22].

**Figure 1:**
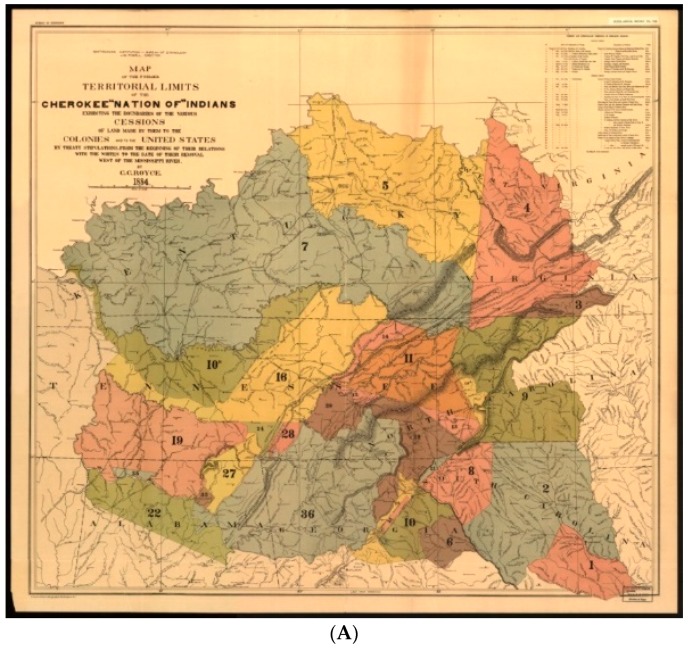

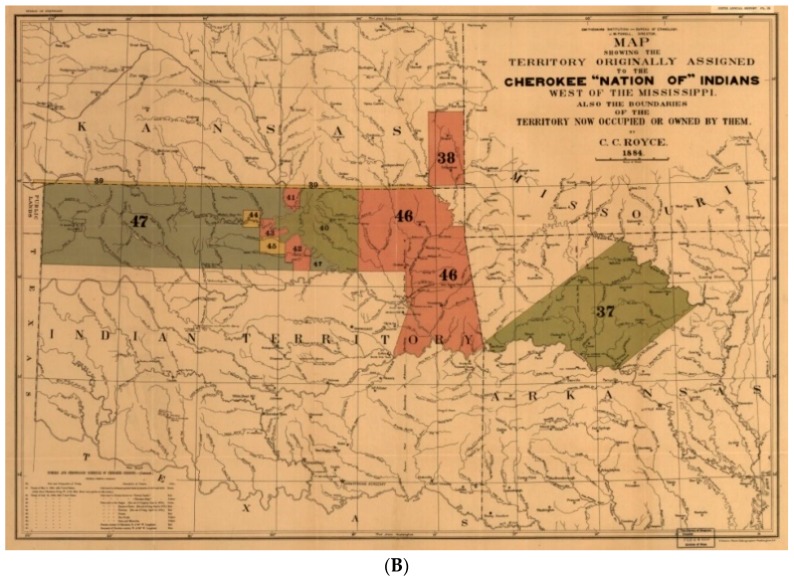
Cherokee territorial lands [26]. (**A**) ′′Map of the former territorial limits of the Cherokee ′Nation of′ Indians′′, i.e., prior to displacement of Euro-Americans. (**B**) ′′Map showing the territory originally assigned Cherokee ′Nation of′ Indians′′, i.e., after the forcible relocation known as the ′′Trail of Tears”.

The Cherokee Native Americans are a tribe of Iroquoian-language people who lived in the southern part of the Appalachian Mountain region in present-day northern Georgia, eastern Tennessee, and western North Carolina and South Carolina at the time of European contact [13] (Figure 1A). During and after the American Revolution, Cherokee wars with European settlers resulted in the surrender of vast amounts of territory. Gold was discovered on Cherokee land in north Georgia and the Treaty of New Echota (1835) ceded all Cherokee land east of the Mississippi River to the United States. Congress passed the Indian Removal Act in 1830, and the forced eviction of as many as 16,000 Cherokee took place during the fall and winter of 1838–1839 to a new territory in north-eastern Oklahoma (Fibure 1B). During this “Trail of Tears”, an estimated one-fourth of the Cherokee died. However, at the time of the removal, a few hundred Cherokee successfully escaped to the mountains of western North Carolina, forming what is now the Eastern Band of Cherokee Indians.

In this review, I have consulted the ethnobotanical sources for plants used in Cherokee traditional medicine [15,16,17,18,19,20,21,22,23,24] and I have carried out a literature search using Google Scholar, PubMed, ResearchGate, and Science Direct for phytochemical analyses on the plant species. Note that in many instances, the phytochemistry was determined by plants not collected in the south-eastern United States; many of the species have been introduced to other parts of the world and some species are native to other continents besides North America. The phytochemistry, therefore, may be affected by the different geographical and climatic conditions [25]. Sources reporting the phytochemical constituents, regardless of geographical origin, have been included.

## 2. Cherokee Aromatic Medicinal Plants and Their Phytochemical Constituents

The plants used by the Cherokee people for traditional medicines for which the phytochemistry has been investigated are summarized in Table 1.

## 3. Cherokee Aromatic Medicinal Plants Currently in Use as Herbal Medicines

### 3.1. Achillea millefolium L.

*Achillea millefolium* (yarrow) is native to temperate regions of the Northern Hemisphere but has been introduced worldwide [510]. The traditional medical uses of *A. millefolium* have been reviewed and the plant has been used since ancient times as a wound-healing agent and to treat gastrointestinal complaints [510,511,512]. Consistent with this, the Cherokee have also used *A. millefolium* as an antihemorrhagic; for healing wounds, treating bloody hemorrhoids and bloody urine, and for bowel complaints [15,17,510]. In addition, infusions of *A. millefolium* have been used as a treatment for fever [15,17,510]. Yarrow extract has shown spasmogenic effects on murine and human gastric antrum, consistent with its traditional use to treat dyspepsia [513]. In a double-blind clinical trial, *A. millefolium* ointment was shown to reduce pain, inflammation, and ecchymosis in episiotomy wound healing [514].

The essential oils of *A. millefolium* have shown wide variation depending on geographical location and growing season. Volatile oil samples from Turkey [48] and Macedonia [51] were dominated by 1,8-cineole and camphor, whereas the essential oil from Lavras, Brazil, was rich in chamazulene [49]. The essential oil from Lithuania showed wide variation in composition depending on morphological type (flower color) as well as plant phenology [50]; γ-terpinene and cadinene (isomer not identified) were the major components during the flowering phase, but β-pinene was abundant during the vegetative phase. Conversely, *A. millefolium* leaf essential oil from Portugal was rich in 1,8-cineole during the flowering phase, but germacrene D dominated the oil during the vegetative phase [53].

The non-volatile chemical components of *A. millefolium* are generally dominated by phenolics (e.g., chlorogenic acid and other quinic acid derivatives) and flavonoids and flavonoid glycosides (e.g., luteolin, apigenin, and quercetin, and their glycosides) [38,39,40,41,42,44,46,47]. Chlorogenic acid has shown in vivo wound-healing properties in rat models [515,516]. Likewise, the flavonoid apigenin [517,518] as well as an apigenin glycoside [519] have shown in vivo wound-healing effects in rodent models. Similarly, luteolin [520,521,522], luteolin-7-*O*-glucoside [523], quercetin [524,525,526] and several quercetin glycosides [527,528,529,530,531] have shown wound-healing effects.

### 3.2. Caulophyllum thalictroides (L.) Michx.

A decoction of the roots of *C. thalictroides* (blue cohosh) has been used by the Cherokee as an anticonvulsive (to treat “fits and hysterics”) and antirheumatic [15]. The plant is also used as a gynecological aid, to promote childbirth and to treat womb inflammation [15]. These traditional uses are in apparent contrast to the observed toxic effects (convulsions, respiratory paralysis) of the plant observed in range animals such as sheep [108]. The rhizome of *C. thalictroides* contains several quinolizidine alkaloids, including *N*-methylcytisine (also known as caulophylline), baptifoline, anagyrine, and lupanine [108,110,112]. *N*-Methylcytisine is known to stimulate the central nervous system, and in high doses causes convulsions followed by paralysis [532]. Acute lupanine toxicity is characterized by neurotoxic effects including decreased cardiac contractility, blocking of ganglionic transmission and contraction of uterine smooth muscle [533]. This latter effect explains the traditional Cherokee use to promote childbirth. Apparently, lupanine, in lower doses, does not exhibit sub-chronic, chronic, reproductive, or mutagenic toxic effects [533]. Both *N*-methylcytisine [110] and anagyrine [534] have been shown to be teratogenic, however. The aporphine alkaloid magnoflorine, on the other hand, has shown sedative and anxiolytic effects [535] and may be responsible for the anti-convulsive and sedative uses of *C. thalictroides* in Cherokee traditional medicine.

Lee and co-workers [115] have shown that the oleanolic acid glycosides caulosides A–D exert anti-inflammatory effects by way of inhibiting expression of inducible nitric oxide synthase (iNOS) and the pro-inflammatory cytokines tumor necrosis factor alpha (TNF-α) and interleukin 6 (IL-6). The anti-inflammatory effects of *C. thalictroides* triterpene saponins are consistent with the Cherokee traditional uses to treat rheumatism and inflammation.

### 3.3. Cimicifuga racemosa (L.) Nutt. (syn. Actaea racemosa L.)

Black cohosh (*C. racemosa*) has been a popular herbal supplement for many years [536]. The plant is reputed to possess anti-inflammatory, diuretic, sedative, and antitussive activities [511], and the root has been reported to have estrogenic activity [537,538,539]. Fukinolic acid [137] and formononetin [511] have been reported to be estrogenic constituents of *C. racemosa* rhizome. The traditional Cherokee use of *C. racemosa* rhizome to stimulate menstruation [15] is consistent with the reported estrogenic activity. There have been conflicting reports regarding the estrogenic activity of *C. racemosa* rhizome, however [540,541,542], and a survey of 13 populations of *C. racemosa* in the eastern United States failed to detect the presence of formononetin [543]. Molecular docking studies have suggested that *C. racemosa* triterpenoids are unlikely estrogen receptor binding agents, but any estrogenic activity of *C. racemosa* extract is probably due to phenolic components such as cimicifugic acid A, cimicifugic acid B, cimicifugic acid G, cimiciphenol, cimiciphenone, cimiracemate A, cimiracemate B, cimiracemate C, cimiracemate D, and fukinolic acid [544]. Although recent evidence suggests the estrogen receptor not to be a target of *C. racemosa* phytochemical constituents, other biomolecular targets may be involved. Rhizome extracts of *C. racemosa* have been shown to interact with the serotonin receptor [545], the μ-opioid receptor [546,547] as well as the γ-aminobutryic acid type A (GABA_A_) receptors [548]. Modulation of these receptors may contribute to some of the biological effects of *C. racemosa* extracts.

Reviews of several randomized clinical trials have failed to demonstrate efficacy of *C. racemosa* on menopausal symptoms [549,550]. However, one randomized, placebo-controlled double-blind clinical trial with menopausal women, concluded that *C. racemosa* extract showed superiority over a placebo in ameliorating menopausal disorders [551]. Clinical studies have generally suggested *C. cimicifuga* use to be safe, but there have been some case reports indicating safety concerns [552].

The Cherokee have also used infusions of *C. racemosa* rhizome to treat rheumatism, coughs, and colds [15]. Aqueous extracts of *C. racemosa* have demonstrated reduction of the release of pro-inflammatory cytokines interleukin-6 (IL-6), tumor necrosis factor alpha (TNF-α), and interferon-gamma (IFN-γ) in whole blood, and the prominent active component responsible was isoferulic acid [553]. The ethyl acetate fraction of the aqueous extract of *C. racemosa* was also shown to suppress the release of TNF-α, due to cimiracemate A [554]. Aqueous extracts reduced inducible nitric oxide synthase (iNOS) protein expression as well as iNOS mRNA levels, but did not inhibit iNOS enzymatic activity; the triterpenoid glycoside 23-*epi*-26-deoxyactein was found to be the active principle in the extract [555]. These effects likely explain the anti-inflammatory activities of *C. racemosa* and their traditional uses to treat rheumatism and other inflammatory diseases.

### 3.4. Hamamelis virginiana L.

*Hamamelis virginiana*, American witch hazel, is a shrub or small tree, native to eastern North America. Several Native American tribes have used the plant for numerous medicinal purposes. Decoctions of the bark or the stems of witch hazel have been used as a topical lotion for cuts, bruises, insect bites, external inflammations, and other skin problems [15]. In addition, the Cherokee people took infusions of witch hazel for periodic pains, to treat colds, sore throats, and fevers. Modern uses of witch hazel include treatment of hemorrhoids, inflammation of the mouth and pharynx (leaf only), inflammation of the skin, varicose veins, wounds and burns [537]. *Hamamelis virginiana* leaves contain up to 10% tannins, including gallic acid, polygalloylglucose, hamamelitannin and analogs, flavonoids, and proanthocyanidins [511], which are responsible for the observed astringent, anti-inflammatory, and hemostatic effects [537]. The bark also contains hamamelitannin and analogs, and proanthocyanidins [511].

The aqueous ethanol extract of *H. virginiana* showed anti-inflammatory activity in the croton oil mouse ear edema test [556] as well as the induced rat paw edema assay, confirming its use as an anti-inflammatory agent [557]. The extract also showed notable antiviral activity against Herpes simplex virus type 1 (HSV-1) [556]. Hamamelitannin and galloylated proanthocyanidins from *H. virginiana* were found to be potent inhibitors of 5-lipoxygenase (5-LOX) [558]. *Hamamelis* proanthocyanidins were found to stimulate cell growth of keratinocytes, enhancing cell growth, and are likely responsible for the dermatological use of tannin-containing witch hazel preparations [559]. *Hamamelis* tannins have also shown cytotoxic activity against HT-29 human colorectal adenocarcinoma cells [223] and antiviral activity against influenza A virus and human papillomavirus [560].

The anti-inflammatory activity of witch hazel was demonstrated in a clinical study using a lotion prepared from *H. virginiana* distillate, which showed suppression of erythema after ultraviolet (UVB) light exposure [561]. Similarly, in a clinical trial with patients suffering from atopic eczema, a cream containing *H. virginiana* distillate significantly reduced skin desquamation, itching and redness [562]. Of course, *H. virginiana* distillate will not contain tannins.

### 3.5. Hydrastis canadensis L.

Goldenseal (*Hydrastis canadensis*), a perennial herb in the Ranunculaceae, is native to eastern North America from Ontario, Canada, south to Alabama and Georgia [563]. The Cherokee used the root decoction of goldenseal as a tonic and wash for local inflammations; took the root decoction orally to treat cancer, dyspepsia, and general debility [15]. Goldenseal is still used in herbal medicine to control muscle spasms, treat cancer, increase blood pressure, treat gastrointestinal disorders, manage painful and heavy menstruation, treat infections topically, and reduce swelling [537,564].

The major components in goldenseal root are isoquinoline alkaloids hydrastine, berberine, and canadine, and berberine likely accounts for the biological activities of goldenseal. Berberine has shown in vitro cytotoxic activity to HeLa human epitheloid cervix carcinoma, SK-OV-3 human ovarian carcinoma, HEp2 human laryngeal carcinoma, HT-29 human colorectal adenocarcinoma, MKN-45 human gastric cancer, HepG2 human hepatocellular carcinoma, MCF-7 and MDA-MB-231 human breast adenocarcinoma cell lines [565,566,567,568]. The cytotoxicity of berberine can be attributed to DNA intercalation [569,570,571] and modulation of the human epidermal growth factor receptor 2 (HER2)/phosphatidylinositol-3-kinase (PI3K)/protein kinase B (Akt) signaling pathway [572,573]. Berberine has also shown antibacterial activity against *Staphylococcus aureus* [238,574], and *Helicobacter pylori* [453]; antiparasitic activity against *Entamoeba histolytica*, *Giardia lamblia*, *Trichomonas vaginalis*, *Trypanosoma brucei*, *Trypanosoma congolense*, *Leishmania braziliensis panamensis*, *Leishmania major*, and *Plasmodium falciparum* [575,576,577,578]; and anti-inflammatory activity in a serotonin-induced mouse paw edema assay [579]. In a randomized, double-blind, placebo-controlled clinical trial with patients suffering from acute watery diarrhea due to cholera, berberine showed a significant reduction in stool volume compared to the placebo [580]. Several clinical studies have demonstrated antihyperlipidemic effects of berberine in humans [581].

### 3.6. Juncus effusus L.

*Juncus effusus* (common rush) is native to North and South America, Europe, Asia, and Africa [563]. There are numerous varieties and subspecies of *J. effusus* with at least two in eastern North America [582]. The Cherokee took a decoction of the plant as an emetic, while an infusion was used to wash babies to strengthen them and prevent lameness [15]. In Chinese Traditional Medicine (TCM), *J. effusus* is used as a sedative, anxiolytic, antipyretic, and to reduce swelling. Extracts of *J. effusus* have revealed several cinnamoylglycerides [252,253], cycloartane triterpenoids [255,256,257], phenanthrenes [258,259,260,261,262,263,264,266,267,269,270,271,272,583,584], and pyrenes [265,268]. Dehydroeffusol, effusol, and juncusol, phenanthrenes isolated from *J. effusus*, have shown anxiolytic and sedative effects in a mouse model [264,271], likely due to modulation of the gamma-amino butyric acid type A (GABA_A_) receptor [272]. The GABA_A_ modulatory activity may account for the TCM use of *J. effusus* as a sedative and anxiolytic agent. Several *J. effusus* phenanthrenes have shown inhibition of NO production in lipopolysaccharide (LPS)-activated murine macrophage RAW 264.7 cells, indicating anti-inflammatory activity [270].

### 3.7. Panax quinquefolius L.

American ginseng (*Panax quinquefolius*) is a member of the Araliaceae and is native to eastern North America [585]. Ginseng root from *P. ginseng* or *P. notoginseng*, has been used for thousands of years in the Asian traditional medicine. *Panax quinquefolius* is currently cultivated in the United States, Canada, and China, and is used as a medical tonic worldwide. Native Americans have used *P. quinquefolius* for numerous medical problems as well as a general tonic [15], and European settlers had also utilized this plant for similar purposes [586]. The Cherokee used the root as an expectorant, to treat colic, oral thrush, and as a general tonic [15].

The phytochemistry and pharmacology of *P. quinquefolius* has been reviewed several times [333,339,341,342]. The major components in *P. quinquefolius* roots are triterpenoid glycosides, the ginsenosides, as well as several polyacetylenes. The ginsenosides have shown anti-inflammatory, antiproliferative, hepatoprotective, cardioprotective, neuroprotective, cholesterol-lowering, and cognitive improvement [340].

Several clinical trials have been carried out using *P. quinquefolius* extracts. In terms of cognitive function, a randomized, double-blind, placebo-controled crossover trial, *P. quinquefolius* extract showed significant improvement in working memory, choice reaction time and “calmness” [587]. A clinical trial to study the effects of *P. quinquefolius* extract on cancer-related fatigue showed a promising significant trend in relieving fatigue [588]. *Panax quinquefolius* extracts were found to be clinically effective in preventing upper respiratory infections in healthy adult senior citizens [589,590].

### 3.8. Sanguinaria canadensis L.

Bloodroot (*Sanguinaria canadensis*, Papaveraceae) is native to eastern North America [591]. The plant has been used by Native Americans as a traditional medicine for a variety of ailments [455]. The Cherokee used a decoction of the root, in small doses, for coughs, lung inflammations, and croup, and a root infusion was used as a wash for ulcers and sores [15]. The roots are rich in isoquinoline alkaloids, including sanguinarine, chelerythrine, sanguilutine, chelilutine, sanguirubine, chelirubine, protopine, and allocryptopine [455]. The traditional Cherokee uses of bloodroot as a cough medicine/respiratory aid as well as for treating ulcers and sores can be attributed to the antimicrobial activities of the isoquinoline alkaloids [592]. Thus, for example, sanguinarine has shown antimicrobial activity against methicillin-resistant *Staphylococcus aureus* (MRSA) [593], biofilm-forming *Candida* spp. [594], *Mycobacterium* spp. [452], and *Helicobacter pylori* [453].

### 3.9. Scutellaria lateriflora L.

Infusions of the roots of blue skullcap (*Scutellaria lateriflora*, Lamiaceae) were used by the Cherokee for monthly periods and to treat diarrhea; root decoctions were used as an emetic to expel afterbirth and to remedy breast pains [15]. Interestingly, the aerial parts, rather than the roots, are currently used as an herbal medicine as an anxiolytic, sedative and antispasmodic [511,537,595,596].

The phytochemistry and pharmacology of *S. lateriflora* have been reviewed [469]. The secondary metabolites from the aerial parts of *S. lateriflora* are dominated by flavonoid glycosides (baicalin, dihydrobaicalin, lateriflorin, ikonnikoside I, scutellarin (scutellarein-7-*O*-glucuronide), and oroxylin A-7-*O*-glucuronide, and 2-methoxy-chrysin-7-*O*-glucuronide), flavonoid aglycones (baicalein, oroxylin A, wogonin, and lateriflorein), phenylpropanoids (caffeic acid, cinnamic acid, *p*-coumaric acid, and ferulic acid), and clerodane diterpenoids (scutelaterin A, scutelaterin B, scutelaterin C, ajugapitin, and scutecyprol A) [469]. The essential oil from the aerial parts of *S. lateriflora* (collected in northern Iran) was composed largely of sesquiterpene hydrocarbons, δ-cadinene (27%), calamenene (15.2%), β-elemene (9.2%), α-cubenene (4.2%), α-humulene (4.2%), and α-bergamotene (2.8%) [470].

The flavonoids scutellarin and baicalin and the phenylpropanoid ferulic acid have shown in vitro estrogenic effects [597,598], and may be responsible for the traditional Cherokee uses of *S. lateriflora*.

Consistent with the current herbal medicinal use of *S. lateriflora*, the plant has shown anti-convulsant activity in rodent models of acute seizures, attributable to the flavonoid constituents [474]. Baicalin has shown anti-convulsant activity in pilocarpine-induced epileptic model in rats [599], and wogonin has shown anti-convulsant effects on chemically-induced and electroshock-induced seizures in rodents [600]. In addition, scutellarin has shown relaxant activity using rodent aorta models [601,602], while wogonin showed smooth muscle relaxant activity in rat aorta [603] and rat uterine smooth muscle [604]. On the other hand, both baicalin and baicalein inhibited NO-mediated relaxation of rat aortic rings [605]. Baicalein and baicalin have shown anxiolytic activity [606]. Apparently, baicalin and wogonin exert their anxiolytic effects through allosteric modulation of the GABA_A_ receptor by way of interaction at the benzodiazepine site [607,608]. Conversely, baicalein promotes anxiolytic effects via interaction with non-benzodiazepine sites of the GABA_A_ receptor [609]. There have apparently been no clinical trials on the root extracts of *S. lateriflora*. However, in randomized, double-blind, placebo-controlled crossover clinical trials, the anxiolytic effects of *S. lateriflora* herbal treatments significantly enhanced overall mood without reducing cognition or energy [610,611].

## 4. Conclusions

This is not a complete list of the phytochemistry of Cherokee aromatic medicinal plants. Numerous plants described in the Cherokee ethnobotanical literature [15,16,17,18,19,20,21,22,23,24] have not been investigated for phytochemical constituents or pharmacological activity. In addition, in many instances the phytochemistry is not sufficiently characterized, particularly in terms of the plant tissues used in Cherokee traditional medicine. In this review, there are numerous instances where the phytochemical constituents and the biological activities associated with them correlate with the traditional Cherokee uses of the plant, but there are several instances where there is no apparent correlation. Therefore, much work is needed to add to our knowledge of the pharmacological properties of the chemical components, not to mention potential synergistic or antagonistic interactions.

## Figures and Tables

**Table 1:** List of Cherokee aromatic medicinal plants, their traditional uses, and phytochemical constituents and biological activities.

Scientific Name	Family	Common Name	Cherokee Use	Part Used	Chemical Constituents and Activities	Ref.
*Acer rubrum* L.	Sapindaceae	Red maple	analgesic (cramps), eye soreness	bark		[15]
					Leaves: 1-*O*-galloyl-α-l-rhamnose, 1-*O*-galloyl-β-d-glucose, gallic acid, methyl gallate, ethyl gallate, *m*-digallate, ethyl digallate	[27]
					Leaves: gallic acid, methyl gallate, ethyl gallate, *m*-digallate, ethyl *m*-digallate, 1-*O*-galloyl-β-d-glucose, 1-*O*-galloyl-α-l-rhamnose, kaempferol 3-*O*-β-d-glucoside, kaempferol 3-*O*-β-d-galactoside, kaempferol 3-*O*-β-l-rhamnoside, kaempferol-3-*O*-rhamnoglucoside, quercetin 3-*O*-β-d-glucoside, quercetin 3-*O*-β-l-rhamnoside and quercetin	[28]
					Leaves: major gallotannins: maplexin B, ginnalin B, ginnalin C, ginnalin A, maplexin F and a pair of isomers, 6-*O*-digalloyl-2-*O*-galloyl-1,5-anhydro-d-glucitol and 2-*O*-digalloyl-6-*O*-galloyl-1,5-anhydro-d-glucitol; ginnalin A was the predominant gallotannin	[29]
					Bark: catechin, epicatechin, epicatechin gallate, procyanidin A_6_, procyanidin A_2_, quercetin-3-*O*-α-l-rhamnopyranoside, quercetin-3-*O*-(3′′-*O*-galloyl)-α-l-rhamnopyranoside, quercetin-3-*O*-(2′′-*O*-galloyl)-α-l-rhamnopyranoside, nortrachelogenin-8′-*O*-β-d-glucopyranoside, 7,8-dihydroxy-6-ethoxycoumarin, phloridzin, methyl vanillate, 3,5-dihydroxy-4-methoxybenzoic acid, and 3-methoxy-4-hydroxyphenol-1-*O*-β-d-(6′-*O*-galloyl)- glucopyranoside	[30]
					Bark: gallotannins, named maplexins A–E; showed α-glucosidase inhibitory activity	[31]
					Bark: gallotannins, maplexins F–I; phenolic glycosides, rubrumosides A–B. The maplexins showed α-glucosidase inhibitory activity	[32]
					Bark: Maplexins C and D showed cytotoxic activity on HCT-116 and MCF-7 cells	[33]
					Leaves and flowers: 2-methoxyl-1-*O*-galloyl-*myo*-inositol, 1-*O*-(3′-methoxyl-galloyl)-β-d-glucose	[34]
*Acer saccharinum* L.	Sapindaceae	Silver maple	analgesic (cramps), eye soreness	bark		[15]
					Leaves: methyl gallate; cytotoxic to B16 melanoma in mice	[35]
					Leaves: glucitol-core containing gallotannins (GCGs), ginnalins A–C, maplexins B, D, and F; phenolics, methyl syringate, methyl gallate, and 3-methoxy-4-hydroxyphenol-1-β-d-(6-galloyl)-glucopyranoside; sesquiterpenoid pubineroid A	[36]
*Achillea millefolium* L.	Asteraceae	Yarrow	hemorrhages (leaves), fever (infusion)	leaves		[15]
					Herb: 5-hydroxy-3,6,7,4′-tetramethoxyflavone, artemetin, casticin	[37]
					Herb: chlorogenic acid, vicenin-2, luteolin-7-*O*-glucoside, rutin, apigenin-7-*O*-glucoside, luteolin, and apigenin	[38]
					Herb: apigenin, luteolin, centaureidin, β-sitosterol, 3β-hydroxy-11α,13-dihydro-costunolide, desacetylmatricarin, leucodin, achillin, 8α-angeloxy-leucodin and 8α-angeloxy-achillin	[39]
					Herb: chlorogenic acid, rutin, luteolin 7-*O*-glucoside, 1,3-dicaffeoylquinic acid, 1,4-dicaffeoylquinic acid, 3,4-dicaffeoylquinic acid, apigenin 4′-*O*-glucoside, apigenin 7-*O*-glucoside, luteolin 4′-*O*-glucoside, 3,5-dicaffeoylquinic acid; luteolin and apigenin 7-*O*-glucoside showed notable antiplasmodial activity	[40]
					Herb: 5-*O*-caffeoylquinic acid, quercetin *O*-hexoside, 3,4-*O*-dicaffeoylquinic acid, quercetin *O*-acetylhexoside, *cis*-3,5-*O*-dicaffeoylquinic acid, *trans*-3,5-*O*-dicaffeoylquinic acid, 4,5-*O*-dicaffeoylquinic acid, apigenin 7-*O*-glucoside, luteolin *O*-acetylhexoside, apigenin *O*-acetylhexoside	[41]
					Herb: chlorogenic acid, 3,5-dicaffeoyl quinic acid, 4,5-dicaffeoyl quinic acid, apigenin 7-*O*-glucoside, luteolin	[42]
					Flowers: methyl achimillate A, methyl achimillate B, methyl achimillate C; all three compounds active against P-388 leukemia in vivo (mouse)	[43]
					Herb: dihydrodehydrodiconiferyl alcohol 9-*O*-β-d-glucopyranoside, apigenin, apigenin-7-*O*-β-d-glucopyranoside, luteolin, luteolin-7-*O*-β-d-glucopyranoside, luteolin-4′-*O*-β-d-glucopyranoside, rutin, 3,5-dicaffeoylquinic acid, and chlorogenic acid; apigenin and luteolin showed in vitro estrogenic activity	[44]
					Herb: hydroalcoholic extract showed antinociceptive activity	[45]
					Herb: rutin, schaftoside, isoschaftoside, luteolin-7-*O*-glucoside (major), apigenin-7-*O*-glucoside (major), luteolin-7-malonylglucoside, apigenin-7-malonylglucoside, luteolin, apigenin	[46]
					Herb: five flavonoids (apigenin, luteolin, centaureidin, casticin and artemetin) and five sesquiterpenoids (paulitin, isopaulitin, psilostachyin C, desacetylmatricarin and sintenin); centaureidin, casticin, and paulitin showed good in vitro cytotoxic activity on HeLa, MCF-7, and A-431 cells	[47]
					Herb EO: 1,8-cineole (24.6%), camphor (16.7%), α-terpineol (10.2%); weak antimicrobial activity on *Streptococcus pneumoniae*, *Clostridium perfringens*, and *Candida albicans*	[48]
					Herb EO: germacrene D (6.1%), chamazulene (48.3%); shows antitrypanosomal activity (*Trypanosoma cruzi*)	[49]
					Herb EO: α-pinene (0.6–10.0%), camphene (0.4–15.4%), β-pinene (1.9–38.7%), limonene (1.4–3.8%), γ-terpinene (3.5–13.1%), β-caryophyllene (4.4–13.8%), germacrene D (1.7–10.7%), cadinene (0.7–32.2%)	[50]
					Herb supercritical CO_2_ extract: myrcene (4.9%), *p*-cymene (5.4%), 1,8-cineole (16.2%), γ-terpinene (9.4%), camphor (38.4%), bornyl acetate (4.3%)	[51]
					Herb EO: β-pinene (4.3%), 1,8-cineole (15.2%), β-cubebene (4.0%), germacrene D (14.1%), τ-cadinol (4.4%)	[52]
					Herb EO: sabinene (5.4%), 1,8-cineole (24.5%), trans-sabinene hydrate (10.2%), cis-sabinene hydrate (4.6%), camphor (4.9%), terpinen-4-ol (5.6%), bornyl acetate (4.0%), germacrene D (7.2%)	[53]
*Aesculus pavia* L.	Sapindaceae	Red buckeye	tumors, infections (poultice of nuts)	nuts		[15]
					Fruits: polyhydroxyoleanene triterpenoid saponins (aesuliosides Ia–Ie, IIa–IId, and IVa–IVc)	[54]
					Fruits: 13 polyhydroxyoleanene pentacyclic triterpenoid saponins, aesculiosides IIe–IIk, and IIIa–IIIf, together with 18 known compounds: aesculiosides Ia–Ie, IIa–IId, IVa–IVc, 3-*O*-[β-d-galactopyranosyl(1→2)]-α-l-arabinofuranosyl(1→3)-β-d-glucuronopyranosyl-21,22-*O*-diangeloyl-3β,15α,16α,21β,22α,28-hexahydroxyolean-12-ene, 3-*O*-[β-d-glucopyranosyl(1→2)]-α-l-arabinofuranosyl(1→3)-β-d-glucuronopyranosyl-21,22-*O*-diangeloyl-3β,16α,21β,22α,24β,28-hexahydroxyolean-12-ene, 3-*O*-[β-d-galactopyranosyl(1→2)]-α-l-arabinofuranosyl(1→3)-β-d-glucuronopyranosyl-21,22-*O*-diangeloyl-3β,16α,21β,22α,28-pentahydroxyolean-12-ene, R_1_-barrigenol, scopolin, and 5-methoxyscopolin. Aesculioside IIc, 3-*O*-[β-d-galactopyranosyl(1→2)]-α-l-arabinofuranosyl(1→3)-β-d-glucuronopyranosyl-21,22-*O*-diangeloyl-3β,15α,16α,21β,22α,28-hexahydroxyolean-12-ene, 3-*O*-[β-d-glucopyranosyl(1→2)]-α-l-arabinofuranosyl(1→3)-β-d-glucuronopyranosyl-21,22-*O*-diangeloyl-3β,16α,21β,22α,24β,28-hexahydroxyolean-12-ene, 3-*O*-[β-d-galactopyranosyl(1→2)]-α-l-arabinofuranosyl(1→3)-β-d-glucuronopyranosyl-21,22-*O*-diangeloyl-3β,16α,21β,22α,28-pentahydroxyolean-12-ene, showed broad cytotoxic activity	[55]
					Fruits: oleane saponins (vaccaroside A, vaccaroside B); showed in vitro cytotoxic activity on FL normal human amniotic cells and A-549 human lung carcinoma cells	[56]
					Leaves: prenylated coumarin pavietin; flavonol glycosides quercetin 3-*O*-α-rhamnoside (quercitrin), quercetin 3-*O*-α-arabinoside, and isorhamnetin 3-*O*-α-arabinoside (distichin). Pavietin showed antifungal activity on *Guignardia aesculi*	[57]
					Leaves: oleane saponins (escins Ia, Ib, IIa, IIb, IIIa)	[58]
					Leaves: oleane saponins (paviosides A–H); all show in vitro cytotoxic activity on J-774 murine macrophage and WEHI-164 murine fibrosarcoma	[59]
*Ageratina altissima* (L.) R.M. King and H. Rob. (syn. *Eupatorium rugosum* Houtt.)	Asteraceae	White snakeroot	fever, tonic, urinary diseases	root		[15]
					Aerial parts: tremetone, 6-hydroxytremetone, dehydrotremetone; tremetone cytotoxic on murine melanoma (B16F_1_) cells	[60]
					Aerial parts: tremetone, dehydrotremetone	[61]
					Aerial parts: tremetone, 6-hydroxytremetone, dehydrotremetone, dehydrotremetone, 2-senecioyl-4-acetylphenol, 2-senecioyl-4-(1-methoxyethyl)phenol, 6-acetyl-2,2-dimethylchroman-4-one, 6-acetyl-7-methoxy-2,2-dimethylchromene, 6-acetyl-8-methoxy-2,2-dimethylchromene, 6-acetyl-5-hydroxy-8-methoxy-2,2-dimethylchromene, 6,7-dimethoxy-2,2-dimethylchromene, and 6-(1-hydroxyethyl)-7-methoxy-2,2-dimethylchromene. Tremetone, hydroxygremetone, dehydrotremetone toxic in goldfish assay	[62]
*Allium canadense* L.	Amarylli-daceae	Meadow garlic	cathartic, diuretic	entire plant		[15]
					Herb: cysteine sulfoxides: methiin, alliin, propiin	[63]
*Allium cernuum* Roth	Amarylli-daceae	Nodding onion	fever	entire plant		[15]
					Herb: diosgenin	[64]
					Herb: cysteine sulfoxides: methiin, alliin, isoalliin	[63]
*Allium tricoccum* Aiton	Amarylli-daceae	Wild leek	tonic (entire plant)	entire plant		[15]
					Herb: methanesulfinothioic acid *S*-methyl ester, methanesulfinothioic acid *S*-2-propenyl ester, 2-propene-1-sulfinothioic acid *S*-methyl ester, methanesulfinothioic acid *S*-(*E*)-1-propenyl ester, methanesulfinothioic acid *S*-(*Z*)-1-propenyl ester, (*E*)-1-propenesulfinothioic acid *S*-methyl ester, 2-propene-1-sulfinothioic acid *S*-2-propenyl ester (allicin), 1-propanesulfinothioic acid *S*-2-propenyl ester, 2-propene-1-sulfinothioic acid *S*-(*E*)-1-propenyl ester, 2-propene-1-sulfinothioic acid S-(*Z*)-1-propenyl ester, (*E*)-1-propenesulfinothioic acid *S*-2-propenyl ester, 1-propanesulfinothioic acid *S*-(*E*)-1-propenyl ester, (*E*)-1-propenesulfinothioic acid *S*-*n*-propyl ester, methyl 1-(methylsulfinyl)propyl disulfide, methyl (*E*)-1-(1-propenylsulfinyl)propyl disulfide, 1-(methylsulfinyl)propyl (*E*,*Z*)-1-propenyl disulfide, methyl 1-(2-propenylsulfinyl)propyl disulfide, 1-(methylsulfinyl) propyl 2-propenyl disulfide, 1-(methylsulfinyl)propyl propyl disulfide, (*E*)-1-propenyl 1-(1-propenylsulfinyl)propyl disulfide, 2-propenyl 1-(2-propenylsulfinyl) propyl disulfide, (*E*)-1-(1-propenylsulfinyl)propyl propyl disulfide, (*E*)-1-propenyl 1-(propylsulfinyl)propyl disulfide, propyl 1-(propylsulfinyl)propyl disulfide	[65]
*Allium vineale* L. ^a^	Amarylli-daceae	Wild garlic	carminative, cathartic, diuretic	entire plant		[15]
					Herb: molluscicidal saponins (nuatigenin 3-*O*-[α-rhamnosyl-(1→2)-β-glucoside, isonuatigenin 3-*O*-[α-rhamnosyl-(1→2)-β-glucoside	[66]
					Herb: diosgenin saponins: diosgenin 3-*O*-α-rhamnosyl-(1→2)-β-glucoside (ophiopogonin C′), diosgenin 3-*O*-β-glucosyl-(1→4)-α-rhamnosyl-(1→4)-β-glucoside, diosgenin 3-*O*-α-rhamnosyl-(1→2)-β-glucosyl-(1→4)-β-glucoside (deltonin), diosgenin 3-*O*-β-glucosyl-(1→4)-α-rhamnosyl-(1→4)-α-rhamnosyl-(1→2)-β-glucoside, diosgenin 3-*O*-β-glucosyl-(1→4)-β-glucosyl-(1→6)-α-rhamnosyl-(1→4)-α-rhamnosyl-(1→2)-β-glucoside, diosgenin 3-*O*-β-glucosyl-(1→3)-β-glucosyl-(1→6)-α-rhamnosyl-(1→4)-α-rhamnosyl-(1→2)-β-glucoside, diosgenin 3-*O*-β-glucosyl-(1→6)-β-glucosyl-(1→4)-α-rhamnosyl-(1→4)-α-rhamnosyl-(1→2)-β-glucoside. Several of these saponins showed molluscicidal activity	[67]
					Herb: flavones: chrysoeriol-7-*O*[2′′-*O*-*E*-feruloyl]-β-d-glucoside, chrysoeriol, isorhamnetin-3-β-d-glucoside, and quercetin	[68]
					Herb EO: methyl (*E*)-1-propenyl disulfide (2.6–12.5%), benzaldehyde (up to 16.4%), dimethyl trisulfide (3.8–17.4%), allyl (*E*)-1-propenyl disulfide (7.9–12.5%), allyl methyl trisulfide (7.9–13.2%), diallyl trisulfide (2.8–10.5%), *p*-vinylguaiacol (5.2–6.5%), 5-methyl-1,2,3,4-tetrathiane (up to 6.1%)	[69]
*Aralia nudicaulis* L.	Araliaceae	Wild sarsaparilla	root infusion taken as a blood tonic	root		[15]
					Rhizome: diacetylenes falcarinol and panaxydol; showed antimycobacterial activity	[70]
*Aralia spinosa* L.	Araliaceae	Devil′s walking stick	root (poisonous) used for emetic, venereal diseases	root		[15]
					Leaf EO: (2*E*)-hexenal (13.8–29.8%), myrcene (13.9–15.1%), β-caryophyllene (8.2–15.7%), α-humulene (1.9–4.9%), germacrene D (28.0–37.3%), (*E*)-nerolidol (1.2–10.4%)	[71]
*Arnica cordifolia* Hook.	Asteraceae	Arnica	pain reliever, anti-inflammatory	flowers		[18]
					Aerial parts: flavonoids: hispidulin, genkwanin, quercetin 3-methyl ether, quercetin 3-gentiobioside, quercetin 3-diglucoside, 6-methoxykaempferol 3-glucoside, isoquercitrin, astragalin, nepitrin, and glucoluteolin	[72]
					Leaves: pseudoguaianolide sesquiterpenoids carabrone, 2,3-dihydroaromaticin, 2,3-dihydroaromatin	[73]
*Artemisia biennis* Willd.	Asteraceae	Biennial wormwood	poultice used on sores and wounds	plant		[15]
					Aerial parts EO: camphor (24.6%), artemisia ketone (11.4%), α-pinene (10.2%), 1,8-cineole (10.1%), germacrene D (5.3%)	[74]
					Aerial parts EO: (*Z*)-β-ocimene (34.7%), (*E*)-β-farnesene (40.0%); EO shows antimicrobial activity	[75]
*Aruncus dioicus* (Walter) Fernald	Rosaceae	Goatsbeard	beaten root applied to bee stings	root	Phytochemistry of Eurasian varieties studied, but not North American varieties	[15]
*Aruncus dioicus* var. *kamtschaticus* (Maxim.) H. Hara ^a^					Aerial parts: aruncin A, aruncin B, aruncide A, aruncide B, aruncide C; aruncin B showed cytotoxic activity on Jurkat T cells	[76]
*A. dioicus* var. *kamtschaticus* ^a^					Aerial parts: aruncin B; cytotoxic to Jurkat T cells (apoptosis, microtubule damage)	[77]
*A. dioicus* var. *kamtschaticus* ^a^					Aerial parts: palmitic acid, 10-nonacosanol, pentacosan-1-ol, phytol, β-sitosterol, β-sitosterol-3-*O*-β-d-glucopyranoside, 2,4-dihydroxycinnamic acid, hyperoside, uridine, and adenosine; β-sitosterol-3-*O*-β-d-glucopyranoside cytotoxic to HL-60 cells; 2,4-dihydroxycinnamic acid and hyperoside showed antioxidant (DPPH radical-scavenging) activity	[78]
*A. dioicus* var. *kamtschaticus* ^a^					Aerial parts: sambunigrin, prunasin, aruncide A, aruncide C, 1-*O*-caffeoyl-β-d-glucopyranose, and caffeic acid; aruncide C cytotoxic to HeLa cells; aruncide A cytotoxic to HL-60 cells; 1-*O*-caffeoyl-β-d-glucopyranose cytotoxic to MCF-7 cells	[79]
*A. dioicus* (Italy)					Young shoots: 4-*O*-caffeoylglucose, chlorogenic acid, dicaffeoylglucose isomer I, dicaffeoylglucose isomer II, 3,5-dicaffeoylquinic acid, prunasin	[80]
*Asarum canadense* L.	Aristolochi-aceae	Wild ginger	vermifuge (root), wounds (poultice of leaves)	root, leaves		[15]
					Leaves: chalcone glycosides (chalcononaringenin 2′,4′-di-*O*-glucoside and chalcononaringenin 2′-*O*-glucoside-4′-*O*-gentiobioside) and flavonol glycosides (quercetin 3-*O*-galactoside, quercetin 3-*O*-robinobioside, quercetin 3-*O*-β-d-galactopyranoside-7-*O*-α-l-rhamnopyranoside, kaempferol 3-*O*-galactoside, kaempferol 3-*O*-glucoside, kaempferol 3-*O*-galactoside-7-*O*-rhamnoside and iso-rhamnetin 3-*O*-rhamnosylgalactoside)	[81]
					Rhizome EO: methyleugenol (44.5%), linalyl acetate (41.1%), geraniol (7.4%), linalool (5.3%)	[82]
					Rhizome EO: linalool (5.0%), linalyl acetate (28.0%), methyleugenol (36.1%)	[83]
					Rhizome EO: methyleugenol (53.6%), linalool (12.5%), α-terpineol (6.6%)	[84]
					Rhizome EO: Linalool (19.4%), α-terpineol (5.9%), methyleugenol (38.5%)	[85]
*Asclepias tuberosa* L.	Apocyn-aceae	Butterfly weed	cough	root		[22]
					Roots: steroids (ascandroside, Δ^5^-calotropin, Δ^5^-calotropin 3′-*O*-β-d-glucoside, Δ^5^-calotropin (3′*S*)-3′-thiazolidinone, Δ^5^-calotropin (3′*R*)-3′-thiazolidinone-*S*-oxide)	[86]
					Roots: Pregnane steroid (ikemagenin, lineolon, pleurogenin) glycosides	[87]
					Aerial parts: Pregnane steroid glycosides (tuberosides A_1_–L_5_)	[88]
					Aerial parts: Pregnane steroid glycosides (tuberosides B_7_ and B_8_)	[89]
					Roots: Pregnane steroid glycosides (tuberosides A_2_, B_1_, B_2_, C_2_, D_1_, D_2_, E_2_, F_2_, G_1_, H_1_, H_2_, I_2_, I_3_, J_3_, K_3_, M_1_, N_1_, O_1_, P_1_, and Q_1_)	[90]
*Baptisia australis* (L.) R. Br.	Fabaceae	Wild indigo	cold infusion purgative/emetic	plant		[15]
					Flavonoids: afrormosin 7-*O*-β-d-glucoside, apigenin 7-*O*-β-d-glucoside, luteolin 7-*O*-β-d-glucoside, formononetin 7-*O*-β-d-glucoside, formononetin, and afrormosin; coumarin trifolirhizin	[91]
					Isoflavonoid: texasin 7-*O*-β-d-glucoside	[92]
					Alkaloids: (+)-sparteine and (–)-*N*-methylcytisine	[93]
*Berberis canadensis* Mill.	Berberi-daceae	American barberry	bark infusion for diarrhea	bark		[15]
					Callus culture: isoquinoline alkaloid jatrorrhizine	[94]
*Betula nigra* L.	Betulaceae	River birch	dysentery, colds	leaves		[15]
					Bud EO: benzyl alcohol (2.4–5.0%), nonanal (0.7–6.6%), eugenol (28.7–55.7%), tricosane (1.6–8.0%), pentacosane (1.3–8.8%), heptacosane (6.2–39.1%)	[95]
					Leaf EO: linalool (9.8–19.2%), eugenol (6.7–13.5%)	[95]
					Bark EO: hexanal (0.8–5.8%), (3*Z*)-hexenol (0–7.8%), *o*-methylanisole (0.3–5.3%), octanoic acid (0.2–7.4%), eugenol (trace-8.8%), decanoic acid (0.6–24.4%), dodecanoic acid (0.7–29.2%), palmitic acid (8.8–43.7%), heptacosane (2.5–24.3%)	[95]
					Bark: betulonaldehyde, lupeol, betulin, betulinic acid, betulin caffeate	[96]
					Buds: combretol, 5-hydroxy-3,4′,7-trimethoxyflavone	[97]
					Buds: 3,5-dihydroxy-4′,7-dimethoxyflavone	[98]
*Callicarpa americana* L.	Lamiaceae	American beautyberry	Alabama tribe of Native Americans (not Cherokee) used a decoction of roots/branches sweat bath for rheumatism, fever	roots, branches		[15]
*Callicarpa americana*					Leaf EO: 1-octen-3-ol (8.5%), β-pinene (8.8%), α-humulene (10.1%), humulene epoxide II (13.9%), intermediol (9.5%), callicarpenal (4.3%); the EO was selectively toxic toward the cyanobacterium *Oscillatoria perornata*	[99]
					Leaf EO: α-humulene, humulene epoxide II, intermediol, callicarpenal; intermediol and callicarpenal showed mosquito repellent activity (*Aedes aegypti*, *Anopheles stephensi*)	[100]
					Leaves: callicarpenal and intermediol; both showed tick repellent activity	[101]
					Fruiting branches: clerodane diterpenoids: 12(*S*),16ξ-dihydroxycleroda-3,13-dien-15,16-olide, 12(*S*)-hydroxy-16ξ-methoxycleroda-3,13-dien-15,16-olide, 12(*S*)-hydroxycleroda-3,13-dien-15,16-olide, 16ξ-hydroxycleroda-3,11(*E*),13-trien-15,16-olide, 3β,12(*S*)-dihydroxycleroda-4(18),13-dien-15,16-olide, and 12(*S*)-hydroxycleroda-3,13-dien-16,15-olide, 16ξ-hydroxycleroda-3,13-dien-15,16-olide, 2-formyl-16ξ-hydroxy-3-A-norcleroda-2,13-dien-15,16-olide. 12(*S*),16ξ-dihydroxycleroda-3,13-dien-15,16-olide, 16ξ-hydroxycleroda-3,11(*E*),13-trien-15,16-olide, 12(*S*)-hydroxycleroda-3,13-dien-16,15-olide, 16ξ-hydroxycleroda-3,13-dien-15,16-olide, 2-formyl-16ξ-hydroxy-3-A-norcleroda-2,13-dien-15,16-olide showed broad-spectrum cytotoxic activity	[102]
*Calycanthus floridus* L.	Calycanth-aceae	Eastern sweetshrub	bark sap used on sores; bark infusion used on hives. Root strong emetic.	bark/root		[15]
					Flowers: anthocyanin pigments: cyanidin-3-glucoside, cyanidin-3-rutinoside	[103]
					Herb EO: α-pinene, 1,8-cineole (major), borneol, bornyl acetate	[104]
					Herb EO: (*E*)-β-ocimene (13.8%)	[105]
*C. floridus* var. *oblongifolius* (Nutt.) Boufford and Spongberg (Iran) ^a^					Floral EO: α-pinene (10.2%), β-pinene (8.6%), 1,8-cineole (33.1%), bornyl acetate (14.1%), α-terpinyl acetate (5.8%), elemol (8.2%)	[106]
*C. floridus* var. *oblongifolius* (Iran) ^a^					Stem EO: α-pinene (10.0%), β-pinene (7.2%), 1,8-cineole (31.7%), bornyl acetate (12.6%), α-terpinyl acetate (6.8%), elemol (9.0%)	[107]
*Caulophyllum thalictroides* (L.) Michx.	Berberi-daceae	Blue cohosh	root decoction given as sedative and anticonvulsive; root taken internally to treat rheumatism	root		[15]
					Roots: alkaloids: *N*-methylcytisine, baptifoline, anagyrine, magnoflorine (major)	[108]
					Roots: quinolizidine alkaloids: *N*-methylcytisine, baptifoline (major), anagyrine	[109]
					Roots: alkaloids: thalictroidine, taspine, magnoflorine, anagyrine, baptifoline, 5,6-dehydro-α-isolupanine, α-isolupanine, lupanine, *N*-methylcytisine, and sparteine; *N*-methylcytisine showed teratogenic activity	[110]
					Roots: piperidine alkaloids (caulophyllumine A, caulophyllumine B), quinolizidine alkaloids (anagyrine, lupanine, *O*-acetylbaptifolin, *N*-methylcytisine), oleanane saponins (caulosides A, B, C, D, G, H, leonticin D, ciwujianoside A, saponin PE)	[111]
					Roots: alkaloids, *O*-acetylbaptifolin, anagyrine, caulophyllumine B, lupanine showed cytochrome-P450 inhibitory activity	[112]
					Roots: oleanane saponins: caulosides A, B, C, D, G; leonticin D, and 3-*O*-β-d-glucopyranosyl-(1→2)-α-l-arabinopyranosyl-echinocystic acid 28-*O*-α-l-rhamnopyranosyl-(1→4)-β-d-glucopyranosyl(1→6)-β-d-glucopyranoside	[113]
					Roots: 22 oleanane saponins; several showed cytotoxicity on HL-60 cells	[114]
					Roots: oleanane saponins caulosides A–D exert anti-inflammatory effects by inhibiting expression of iNOS and proinflammatory cytokines	[115]
*Ceanothus americanus* L.	Rhamnaceae	New Jersey tea	root infusion taken for ′′bowel complaints′′	root		[15]
					Root bark: peptide alkaloids (ceanothine A, B, C; ceanothamine A, B)	[116]
					Root bark: peptide alkaloid americine	[117]
					Root bark: peptide alkaloids (ceanothine D, E; frangulanine, adouetine-X, adouetine-Y)	[118]
*Cercis canadensis* L.	Fabaceae	Redbud	bark infusion used for severe coughs	inner bark		[15]
					Bark EO: 1-hexanol (23.3%), hexanoic acid (18.2%), (2*E*)-hexenoic acid (3.4%)	[119]
*Chelone glabra* L.	Plantagin-aceae	Balmony	herb used to treat skin problems; herb infusion taken as a digestive tonic	herb		[22]
					Leaves: iridoid glycoside catalpol	[120]
*Cichorium intybus* L. ^a^	Asteraceae	Chickory	infusion of root as tonic	root		[15]
					Sesquiterpene lactones (8-deoxylactucin, lactucin, lactupicrin)	[121]
					Leaves and roots: sesquiterpene lactones (lactucin, 11β,13-dihydrolactucin, jacquinelin, 8-desoxylactucin, lactucopicrin, crepidiaside B, loliolide), p-hydroxyphenylacetic acid methy and ethyl esters, cichoriside B, sonchuside A, ixerisoside D, magnolialide	[122]
					Root: sesquiterpene lactones (lactucin, lactucopicrin)	[123]
					Leaves and roots: sesquiterpene lactones (guaianolides, lactucin, lactucopicrin, 11β,13-dihydrolactucin)	[124]
					Flowers: anthocyanin pigments: delphinidin 3,5-di-*O*-(6-*O*-malonyl-β-d-glucoside) and delphinidin 3-*O*-(6-*O*-malonyl-β-d-glucoside)-5-*O*-β-d-glucoside; delphinidin 3-*O*-β-d-glucoside-5-*O*-(6-*O*-malonyl-β-d-glucoside) and delphinidin 3,5-di-*O*-β-d-glucoside	[125]
*Cimicifuga racemosa* (L.) Nutt. (syn. *Actaea racemosa* L.)	Ranuncu-laceae	Black cohosh	root used to stimulate menstruation; root infusion used for rheumatism, coughs, colds	root		[15]
					Rhizome: triterpene glycosides (actein, 27-deoxyactein, cimicfugoside M, and cimicifugoside)	[126]
					Rhizome: triterpene glycosides (cimiaceroside A, 25-*O*-methylcimigenol-3-*O*-β-d-xylopyranoside, 27-deoxyactein, 23-*O*-acetylshengmanol-3-*O*-β-d-xylopyranoside, 16β,23;22β,25-diepoxy-12β-acetoxy-3β,23,24β-trihydroxy-9,19,cyclolanost-7-ene-3-*O*-β-d-xylopyranoside)	[127]
					Rhizome: triterpene glycosides (12β-acetoxycimigenol-3-*O*-β-d-xylopranoside, 25-acetylcimigenol xyloside, cimigenol-3-*O*-β-d-xylopyranoside, acetin, 27-deoxyacetin, cimicifugoside H-1, 23-*O*-acetylshengmanol 3-*O*-β-d-xylopranoside, foetidinol-3-*O*-β-xyloside, cimicifugoside H-2, 25-*O*-methylcimigenol xyloside, 21-hydroxycimigenol-3-*O*-β-d-xylopyranoside, 24-*epi*-7,8-didehydrocimigenol-3-xyloside, cimidahurinine, cimidahurine, and cimifugin)	[128]
					Rhizome: triterpene glycosides (cimiracemosides A–H, 27-deoxyactein, 26-deoxycimicifugoside, actein, acetyl shengmanol xyloside, cimicifugoside (cimigenol-3-*O*-β-d-xylopyranoside), cimiaceroside A, 12β-hydroxycimigenol-3-*O*-β-d-xylopyranoside, and 12β-hydroxycimigenol-3-*O*-α-l-arabinopyranoside)	[129]
					Rhizome: triterpene glycosides (cimigenol 3-*O*-α-l-arabinopyranoside, 25-*O*-methoxycimigenol 3-*O*-α-l-arabinopyranoside, 12β-hydroxycimigenol 3-*O*-α-l-arabinopyranoside, 27-deoxyactein, actein, cimiracemoside F, cimiracemoside G, cimiracemoside H, 25-*O*-acetyl-12β-hydroxycimigenol 3-*O*-α-l-arabinopyranoside, 12β,21-dihydroxycimigenol 3-*O*-α-l-arabinopyranoside, 23-*O*-acetylshengmanol 3-*O*-α-l-arabinopyranoside, (22*R*,23*R*,24*R*)-12β-acetyloxy-16β,23:22,25-diepoxy-23,24-dihydroxy-9,19-cyclolanostan-3β-yl α-l-arabinopyranoside)	[130]
					Rhizome: triterpene glycosides (cimiracemoside H, 26-deoxyactein, 23-*O*-acetylshengmanol 3-*O*-β-d-xylopyranoside, actaeaepoxide 3-*O*-β-d-xylopyranoside, 25-*O*-acetylcimigenol 3-*O*-α-l-arabinopyranoside, 25-*O*-acetylcimigenol 3-*O*-β-d-xylopyranoside)	[131]
					Rhizome: triterpene glycosides (actein, 23-epi-26-deoxyactein, 23-*O*-acetylshengmanol-3-*O*-β-d-xylopyranoside, cimiracemoside D, 25-*O*-acetylcimigenol-3-*O*-β-d-xylopyranoside, and cimigenol)	[132]
					Rhizome: triterpene xyloside, 9,10-seco-9,19-cyclolanostane xyloside (cimipodocarpaside)	[133]
					Rhizome: triterpene xylosides (cimigenol xyloside, 26-deoxyactein, cimicifugoside H-1, and 24-acethylhydroshengmanol xyloside)	[134]
					Rhizome: triterpene xylosides (isocimipodocarpaside, 23-*epi*-26-deoxycimicifugoside, 23-*epi*-26-deoxyactein, 25-anhydrocimigenol xyloside, 23-*O*-acetylshengmanol xyloside, 25-*O*-acetylcimigenol xyloside, 3′-*O*-acetylcimicifugoside H-1)	[135]
					Rhizome: Cimicidol-3-*O*-β-d-xyloside (slightly hepatotoxic)	[136]
					Rhizome: fukiic and piscidic acid esters: (2-*E*-caffeoylfukiic acid (fukinolic acid), 2-*E*-feruloylfukiic acid (cimicifugic acid A), 2-*E*-isoferuloylfukiic acid (cimicifugic acid B), 2-*E*-feruloylpiscidic acid (cimicifugic acid E) and 2-*E*-isoferuloylpiscidic acid (cimicifugic acid F), free caffeic, ferulic and isoferulic acids)	[137]
					Rhizome: phenylpropanoid esters (cimicifugic acid D, petasiphenone, cimiciphenol, cimiciphenone	[138]
					Rhizome: phenylpropanoid esters (cimiracemates A–D)	[139]
					Rhizome: phenylpropanoids (caffeic acid, isoferulic acid, ferulic acid), triterpene xylosides (cimicifugoside H-1, cimiracemoside A, cimicifugoside H-2, (26*R*)-actein, 26-deoxycimicifugoside, (26*S*)-actein, 23-*epi*-26-deoxyactein, 23-acetoxy-shengmanol-3-*O*-β-d-xyloside, 26-deoxyactein, 25-acetoxy-cimigenol-3-*O*-α-l-arabinoside, 25-acetoxy-cimigenol-3-*O*-β-d-xyloside, cimigenol-3-*O*-α-l-arabinoside, cimigenol-3-*O*-β-d-xyloside)	[140]
					Rhizome: polyphenolics (actaealactone, cimicifugic acid G, protocatechuic acid, protocatechualdehyde, p-coumaric acid, caffeic acid, methyl caffeate, ferulic acid, ferulate-1-methyl ester, isoferulic acid, 1-isoferuloyl-â-d-glucopyranoside, fukinolic acid, and cimicifugic acids A, B, and D–F)	[141]
					Rhizome: alkaloids (cyclocimipronidine, cimipronidine methyl ester, cimipronidine, dopargine, salsolinol, 3-hydroxytyrosol 3-*O*-glucoside)	[142]
*Collinsonia canadensis* L.	Lamiaceae	Heal-all	decoction taken as emetic	leaves		[15]
					Leaf EO: germacrene D (46.0%), β-caryophyllene (5.3%), elemicin (3.6%), β-elemene (3.3%)	[143]
					Roots: triterpene glycosides, hederagenin-3-*O*-α-l-arabinopyranoside (leontoside A), 3-*O*-α-l-arabinopyranosylcollinsogenin (collinsonin), 3-*O*-β-d-glucopyranosyl-(1′′→3′)-α-l-arabinopyranosylhederagenin (collinsonidin)	[144]
					Leaf and stem exudates: flavonoids, 2,5-dihydroxy-6,7-dimethoxyflavanone, baicalein-6,7-dimethyl ether, norwogenin-7,8-dimethyl ether, and tectochrysin (5-hydroxy-7-methoxyflavone)	[145]
*Conyza canadensis* (L.) Cronquist (syn. *Erigeron canadensis* L.)	Asteraceae	Horseweed	leaves used for toothache	leaves		[21]
			decoction of herb used to treat diarrhea	herb		[23]
			Mikasuki and Seminole Native Americans used the plant to treat sore throats and respiratory complaints			[146]
					Whole plant: β-sitosterol, stigmasterol, β-sitosterol 3-*O*-β-d-glucoside, harmine, and sphingolipid	[147]
					Whole plant: sphingolipids, 1,3,5-trihydroxy-2-hexadecanoylamino-(6*E*,9*E*)-heptacosdiene, 1,3,5-trihydroxy-2-hexadecanoylamino-(6*E*,9*E*)-heptacosdiene-1-*O*-glucopyranoside, 1,3-dihydroxy-2-hexanoylamino-(4*E*)-heptadecene; p-hydroxybenzoic acid, 3,5-dihydroxybenzoic acid, 3,5-dimethoxybenzoic acid, 3β-hydroxyolean-12-en-28-oic acid, and 3β-erythrodiol	[148]
					Aerial parts: triterpenoid erigeronol (showed potent anti-melanoma cytotoxicity)	[149]
					Whole plant: conyzolide, conyzoflavone (both showed antimicrobial activities)	[150]
					Whole plant: 8*R*,9*R*-dihydroxymatricarine methyl ester, matricarine methyl ester, matricarine lactone, 3β,16β,20β–tritrihydroxytaraxast-3-*O*-palmitoyl ester, friedelin, friedelinol, β-sitosterol, α-spinasterol, 3-isopropenyl-6-oxoheptanoic acid, 9-hydroxy-10*Z*,12*E*-octadecenoic acid, (+)-hydroxydihydroneocarvenol, 3*′*,4*′*,5,7-tetrohydroxydihydroflavone, 9,12,13-trihydroxy-10(*Z*)-octadecenoic acid	[151]
					Whole plants: phenylprobanoyl esters (*rel*-(1*S*,2*R*,3*R*,5*S*,7*R*)-methyl 7-caffeoyloxymethyl-2-hydroxy-3-feruloyloxy-6,8-dioxabicyclo[3.2.1]octane-5-carboxylate, *rel*-(1*S*,2*R*,3*R*,5*S*,7*R*)-methyl 7-feruloyloxymethyl-2-hydroxy-3-feruloyloxy-6,8-dioxabicyclo[3.2.1]octane-5-carboxylate, and *rel*-(1*R*,2*R*,3*R*,5*S*,7*R*)-methyl 7-feruloyloxymethyl-2-feruloyloxy-3-hydroxy-6,8-dioxabicyclo[3.2.1]octane-5-carboxylate)	[152]
					Aerial parts: enyne derivatives, (2*Z*,8*Z*)-matricaria acid methyl ester, (4*Z*,8*Z*)-matricaria lactone, and (4*Z*)-lachnophyllum lactone	[153]
					Aerial parts: (4*Z*)-lachnophyllum lactone, (4*Z*,8*Z*)-matricaria lactone, (2*Z*,8*Z*)-matricaria acid methyl ester; (4*Z*)-lachnophyllum lactone and (4*Z*,8*Z*)-matricaria lactone showed antifungal activity against *Aspergillus niger*, *Cladosporium* sp., and *Penicillium digitatum*	[154]
					Flowering parts: polyphenolic-polysaccharide (anticoagulant, antiplatelet activity)	[155]
					Roots: dihydropyranones conyzapyranone A and B; 4*Z*,8*Z*-matricaria-γ-lactone, 4*E*,8*Z*-matricaria-γ-lactone, 9,12,13-trihydroxy-10(*E*)-octadecenoic acid, epifriedelanol, friedelin, taraxerol, simiarenol, spinasterol, stigmasterol, β-sitosterol, and apigenin; conyzapyranone B, 4*E*,8*Z*-matricaria-γ-lactone, and spinasterol showed cytotoxic activity	[156,157]
					Roots: triterpenoid 3β-erythrodiol (inhibits MKN-45 gastric cell proliferation)	[158]
					Roots: salicylic acid, methyl gallate	[159]
					Roots: lanostane triterpenoids conyzagenin-A, conyzagenin-B	[160]
					Aerial parts EO: limonene (76.0%), α-santalene (5.8%), δ-3-carene (3.9%), myrcene (3.6%)	[161]
					Aerial parts EO: limonene (57.9–81.1%), (*E*)-β-ocimene (0.7–9.1%), *trans*-α-bergamotene (5.6–8.9%), (*Z*)-β-farnesene (tr-11.1%).	[162]
					Aerial parts EO: limonene (50.0–70.3%) and (*E*)-β-ocimene (4.0–7.5%)	[163]
					Aerial parts EO: limonene (70.0%), *trans*-α-bergamotene (7.0%)	[164]
					Aerial parts EO: limonene (77.7–89.4%), *trans*-α-bergamotene (1.5–3.8%), β-pinene (0.8–6.6%), carvone (0.5–1.8%)	[165]
					Aerial parts EO: (*E*)-β-Farnesene (14.6%), spathulenol (14.1%) and limonene (12.3%)	[166]
					Aerial parts EO: limonene (31.2%), camphene (14.2%) and germacrene D (11.3%)	[167]
					Aerial parts EO: limonene (68.3%), δ-3-carene (15.9%)	[168]
					Root EO: (2*Z*,8*Z*)-matricaria ester (88.2–93.9%)	[169]
*Coreopsis tinctoria* Nutt.	Asteraceae	Tickseed	root tea for diarrhea	root		[15]
					Plant: polyacetylenes, (2*S*)-(3*Z*,11*E*)-decadiene-5,7,9-triyne-1,2-diol and (2*R*)-(3*E*,11*Z*)-decadiene-5,7,9-triyne-1,2-diol	[170]
					Plant: seven compounds made up the major contributions of antioxidant activity in *C. tinctoria*, including okanin, isookanin, marein, flavanomarein, 5,7,3′,5′-tetrahydroxyflavanone-7-*O*-glucoside, 3,5-dicaffeoylquinic acid, and chlorogenic acid	[171]
					Flowers: C_14_ polyacetylene glycosides coreosides A–D	[172]
					Buds: C_14_ polyacetylene glycosides coreosides E and F	[173]
					Flowers: C_14_ polyacetylene glycosides coreosides A, B, D, and E	[174]
					Flowers: chalcone marein, flavanone flavanomarein	[175]
					Flowers: chalcone okanin-4′-*O*-β-(6′′-*O*-malonyl)glucopyranoside; flavonoids flavanomarein okanin-4′-*O*-β-d-glucopyranoside, quercetagitin 7-*O*-β-d-glucopyranoside, (2*R*,3*R*)-dihydroquercetin 7-*O*-β-d-glucopyranoside, okanin, quercetin, butein, 2*S*-3′,4′,7,8-tetrahydroxyflavanone, (2*R*,3*R*)-3,3′,5,5′,7-pentahydroxyflavanone, (2*R*,3*R*)-3,4′,5,6,7-penta-hydroxyflavanone, and 2*S*-3′,5,5′,7-tetrahydroxy-flavanone	[176]
					Flowers: flavonoids (flavanomarein, flavanokanin, quercetagitin-7-*O*-glucoside, marein)	[177]
					Flowers: flavonoids ((+)-catechin, kaempferol-3-*O*-d-glycoside, quercetin-3-*O*-glycoside, quercetin-3-*O*-rutinoside	[178]
					Flowers: flavonoids (taxifolin, taxifolin-7-*O*-β-d-glucopyranoside, isookanin, flavanomarein, quercetagetin-7-*O*-β-d-glucopyranoside, 5,7,3′,5′-tetrahydroxyflavanone-7-*O*-β-d-glucopyranoside), chalcones (okanin, marein), and phenolic acids (chlorogenic acid, 3,5-di-*O*-caffeoylquinic acid, 4,5-di-*O*-caffeoylquinic acid)	[179]
					Flowers: quercetagitin-7-*O*-glucoside, marein (major), 1,3-dicaffeoylquinic acid, okanin, acetylmarein	[180]
					Flowers: taxifolin-7-*O*-glucoside, flavanomarein, quercetagetin-7-*O*-glucoside, okanin 4′-*O*-glucoside, okanin, chlorogenic acid	[181]
					Flowers: chlorogenic acid, (*R*/*S*)-flavanomarein, butin-7-*O*-β-d-glucopyranoside, isookanin, taxifolin, 5,7,3′,5′-tetrahydroxyflavanone-7-*O*-β-d-glucopyranoside, marein, and okanin	[182]
					Fruits: flavonoids (marein, flavanomarein, quercetagetin-7-*O*-glucoside, okanin aurone, leptosidin, luteolin, apigenin) and phenolic acids (chlorogenic acid, caffeic acid)	[183]
					Floral EO: limonene (11.3%), α-bergamotene (7.3%)	[184]
*Cornus florida* L.	Cornaceae	Dogwood	bark chewed for headache	bark		[15]
			bark decoction used for fevers, body aches; bark poultice used on sores/ulcers	bark		[22]
					Bark: saponins (sarsapogenin-*O*-β-d-xylopyranosyl-(1→2)-β-d-galactopyranoside and sarsapogenin-*O*-β-d-glucopyranosyl-(1→2)-β-d-galactopyranoside)	[185]
*Datura stramonium* L.	Solanaceae	Jimson weed	leaf poultice applied to boils; leaves smoked for asthma	leaves		[15]
					Root culture: tropane alkaloid (−)-hyoscyamine	[186]
					Root culture: tropane alkaloids (hyoscyamine and scopolamine)	[187]
					Seeds: tropane alkaloid (−)-hyoscyamine	[188]
					Leaves: tropane alkaloids (hyoscyamine and scopolamine)	[189]
*Diospyros virginiana* L.	Ebenaceae	Persimmon	bark infusion for venereal diseases, sore throat and mouth; syrup for oral thrush, bloody discharge from bowels	bark		[17]
					Bark: binaphthoquinone isodiospyrin	[190]
					Fruits: polyphenolics (methyl gallate, gallic acid, luteolin, quercetin, myricetin, yricetin 3-*O*-α-rhamnoside, myricetin 3-*O*-β-glucoside, myricetin 3-*O*-β-glucuronide)	[191]
					Roots: 4-hydroxy-5,6-dimethoxynaphthalene-2-carbaldehyde, 12,13-didehydro-20,29-dihydrobetulin, 7-methyljuglone, diospyrin, isodiospyrin, shinanolone, lupeol, betulin, betulinic acid, betulinaldehyde, and ursolic acid	[192]
*Epilobium angustifolium* L.	Onagraceae	Fireweed	eye conditions due to asthma, allergies	herb		[18]
					Herb: quercetin 3-*O*-(6′′-galloyl)-galactoside, kaempferol 3-*O*-(6′-p-coumaroyl)-glucoside, quercetin 3-*O*-glucuronide, oenothein B; oenothein B inhibited the endopeptidases neutral endopeptidase (NEP) and angiotensin converting enzyme (ACE)	[193]
					Herb: oenothein B (a dimeric macrocyclic ellagitannin) inhibits proliferation of SK-N-SK and PC-3 cells	[194]
					Herb: oenothein B enhances IFNγ production by lymphocytes	[195]
					Herb: ellagitannins (oenothein B, oenothein A, tetramer, pentamer, hexamer, heptamer)	[196]
					Flowers and leaves: ellagitannins (oenothein B, oenothein A, tetramer, pentamer, hexamer, heptamer)	[197]
*Equisetum hyemale* L.	Equiset-aceae	Horsetail	infusion taken for kidneys	plant		[15]
					Stems: (*E*)-feruloyl-4-β-glucoside, (*Z*)-feruloyl-4-β-glucoside, (*E*)-caffeoyl-3-β-glucoside, kaempferol-3-sophoroside, Kaempferol-3-sophoroside-7-β-glucoside, herbacetin-3-sophoroside-8-β-glucoside	[198]
					Aerial parts: 2-(sophorosyl)-1-(4-hydroxyphenyl)ethenone	[199]
*Eryngium yuccifolium* Michx.	Apiaceae	Baneberry, Rattlesnake master	remedy for snakebites	root		[15,17]
			remedy for snakebites		Plant extracts showed inhibition of *Crotalus* proteases	[200]
			urinary-tract inflammation modulator	root		[201]
					Aerial parts EO: polyacetylenes (falcarinone, falcarinol, yuccifolol, 1,8-heptadecadiene-4,6-diyne-3,9-diol)	[202]
					Leaf EO: α-pinene (7.6%), terpinolene (17.8%), β-caryophyllene (6.2%), germacrene D (18.3%), bicyclogermacrene (8.8%), falcarinol (9.6%)	[202]
					Root EO: α-pinene (4.7%), terpinolene (25.8%), 2,3,6-trimethylbenzaldehyde (13.9%), *trans*-β-bergamotene (18.6%)	[202]
					Whole plant: triterpenoid saponins (eryngiosides A–L, saniculasaponin III); flavonoid (kaempferol) glycosides; polyphenolics (caffeates)	[203]
					Root: Triterpenoid saponins	[204]
					The phytochemistry of *E. yuccifolium* has been reviewed	[205]
*Erythronium americanum* Ker Gawl.	Liliaceae	Troutlily	leaves crushed and juice poured over wounds	leaves		[15]
					α-Methylenebutyrolactone	[206]
*Eupatorium maculatum* L.	Asteraceae	Joe-Pye weed	root infusion for kidney, dropsy	root		[15]
					Roots: pyrrolyzidine alkaloids (echinatine, trachelanthamidine)	[207]
					Leaves: pyrrolizidine alkaloid (lycopsamine) and guaianolide sesquiterpene lactone (cumambrin B)	[208]
*Eupatorium perfoliatum* L.	Asteraceae	Boneset	infusion of the plant taken as a tonic, for colds, sore throat, and influenza	plant		[15]
					Aerial parts: guianolide and germacranolide sesquiterpene lactones; flavonoids (eupafolin, hispidulin, patuletin, and kaempferol)	[209]
					Aerial parts: guianolide and germacranolide sesquiterpene lactones	[210]
					Leaves: polyphenolics (protocatechuic acid, hyperoside, quercetin, rutin)	[211]
					Aerial parts: extracts show anti-inflammatory activity, but not immunostimulatory activity	[212]
					Aerial parts: caffeic acid derivatives (5-caffeoylquinic acid (chlorogenic acid), 3-caffeoylquinic acid (neochlorogenic acid) and 3,5-dicaffeoylquinic acid, 2,5-dicaffeoylglucaric acid, 3,4-dicaffeoylglucaric acid, and 2,4- or 3,5-dicaffeoylglucaric acid)	[213]
					Aerial parts: glycosides of kaempferol and quercetin; quaianolides	[214]
					Aerial parts EO: (*E*)-Anethole (16.5%), carvone (7.6%), selin-11-en-4α-ol (5.5%)	[215]
*Fagus grandifolia* Ehrh.	Fagaceae	American beech	nuts chewed for worms	nuts		[15]
					Bark: monolignols [(*Z*)-coniferyl alcohol, (*Z*)-sinapyl alcohol, (*Z*) coniferin, (*Z*)-isoconiferin, (*Z*)-syringin]	[216]
*Frasera caroliniensis* Walter	Gentian- aceae	American Columbo	root used to treat dysentery	root		[15]
					Root: xanthones (1-hydroxy-2,3,4,7-tetramethoxyanthone, 1-hydroxy-2,3,4,5-tetramethoxyxanthone, 1-hydroxy-2,3,7-trimethoxyxanthone, 1-hydroxy-2,3,5-trimethoxyxanthone, swerchirin, 1,3-dihydroxy-4,5-dimethoxyxanthone)	[217]
					Plant: iridoid (loganic acid), secoiridoid (gentiopicroside), and xanthones [1,3-diOH-4,5-diMeO-xanthone, 1-OH-2,3,5-triMeO-xanthone, 1-OH-2,3,4,5-tetraMeO-xanthone, 1-OH-2,3,4,7-tetraMeO-xanthone, 1,8-diOH-3,5-diMeO-xanthone (swerchirin)]	[218]
*Fraxinus americana* L.	Oleaceae	American ash	tonic of inner bark taken for liver and stomach problems	bark		[15]
					Bark: oleoside, syringin, hydroxypinoresinol glycoside, verbascoside, ligustroside	[219]
					Leaves: secoiridoid glucosides (demethylligstroside, (2″*R*)-2″-hydroxyoleuropein, (2″*S*)-2″-hydroxyoleuropein, fraxamoside, frameroside, oleoside dimethyl ester, oleuropein, ligstroside, nuezhenide, (2″*R*)-2″-methoxyoleuropein, (2″*S*)-2″-methoxyoleuropein)	[220]
					Seeds: catechins (epicatechin, catechin-3-*O*-gallate, epigallocatechin, epigallocatechin-3-*O*-gallate, epigallocatechin-(4β-8)-epicatechin, epicatechin-3-*O*-gallate-(4β-8)-epigallocatechin-3-*O*-gallate), procyanidins (procyanidin B-1, procyanidin B-3)	[221]
*Geranium maculatum* L.	Geraniaceae	Wild geranium	cuts, sores, oral thrush	plant		
					Plant EO: citronellol (38%), geraniol (16%), citronellyl formate (10.4%), and linalool (6.45%)	[222]
*Hamamelis virginiana* L.	Hamameli-daceae	Witch hazel	bark infusion used on sores	bark		[15]
					Bark: hamamelitannin cytotoxic to HT-29 colon tumor cells	[223]
					Leaves: gallotannins (hydrolyzable tannins: monogalloyl, heptagalloyl, octagalloyl, and nonagalloyl hexoses), caffeoylquinic acids (3-, and 5-), kaempferol glycoside	[224]
					Bark: polymeric proanthocyanidins (condensed tannins).	[225]
					Bark: tannins, antioxidant, cytotoxic to SK-Mel-28 melanoma cells	[226]
					Bark: condensed (proanthocyanidins) and hydrolyzable (galloylhamameloses) tannins	[227]
*Helenium autumnale* L.	Asteraceae	Sneezeweed	root infusion used to prevent menstruation after childbirth; dried leaves used to induce sneezing	roots, leaves	Apparently the root extract has not been examined	[15]
					Aerial parts: dihydromexicanin E	[228]
					Aerial parts: flexuosin A	[229]
					Aerial parts: helenalin	[230]
					Aerial parts: tenulin	[231]
					Helenalin is cytotoxic (human epithelial type 2, HEp-2, cells)	[232]
					Whole plant: carolenin and carolenalin	[233]
					Flowers: helenalin, autumnolide, mexicanin I; helenalin is cytotoxic	[234]
					Plant: dihydroflorilenalin	[235]
					Plant: 4-*O*-tigloyl-11,13-dihydroautumnolide	[236]
*Hydrastis canadensis* L.	Ranuncu-laceae	Goldenseal	sedative, anti-inflammatory; sores, wounds, cancer	root		[15]
					Rhizomes: alkaloids (berberine, 8-oxotetrahydrothalifendine, canadine, and β-hydrastine); berberine shows antitubercular activity	[237]
					Rhizomes: berberine alkaloids (berberine, β-hydrastine, canadine and canadaline); berberine is antibacterial.	[238]
					Rhizomes: alkaloids (berberine, canadaline, canadine, β-hydrastine, and isocorypalmine)	[239]
					Rhizomes: alkaloids (hydrastinine, hydrastine, canadaline, berberine, canadine)	[240]
					Leaves: 3,4-dimethoxy-2-(methoxycarbonyl)benzoic acid, 3,5,3′-trihydroxy-7,4′-dimethoxy-6,8-*C*-dimethyl-flavone, (±)-chilenine, (2*R*)-5,4′-dihydroxy-6-*C*-methyl-7-methoxy-flavanone, 5,4′-dihydroxy-6,8-di-*C*-methyl-7-methoxy-flavanone, noroxyhydrastinine, oxyhydrastinine, 4′,5′-dimethoxy-4-methyl-3′-oxo-(1,2,5,6-tetrahydro-4*H*-1,3-dioxolo-[4′,5′:4,5]-benzo[1–e]-1,2-oxazocin)-2-spiro-10-phtalan	[241]
					Leaves: flavonoids (sideroxylin, 8-desmethyl-sideroxylin, and 6-desmethyl-sideroxylin); inhibit N or A multidrug resistance pump; synergistic antibacterial activity with berberine	[242]
*Hypericum gentianoides* (L.) Britton, Sterns and Poggenb.	Hyperic-aceae	St. John′s wort	root poultice used for stakebite	root		[15]
					Aerial parts: acyl-phloroglucinols (saroaspidin A, uliginosin A, hyperbrasilol C)	[243]
					Aerial parts: acyl-phloroglucinols (3′-prenyl-phlorisobutyrophenone, saroaspidin A, uliginosin A, hyperbrasilol C)	[244]
					Aerial parts: chlorogenic acid, hyperoside, isoquercitrin, quercitrin, quercetin, at least 9 acyl-phloroglucinols (not identified). The acyl-phloroglucinols fraction reduced prostaglandin E2 synthesis in mammalian macrophages	[245]
*Hypericum hypericoides* (L.) Crantz	Hypericaceae	St. John′s wort	root poultice used for stakebite	root		[15]
					Roots: prenylated benzophenones (clusianone, 7-*epi*-clusianone, 18-hydroxy-7-*epi*-clusianone, 18-hydroxyclusianone, and 18-hydroxyhyperibone K)	[246]
*Iris versicolor* L.	Iridaceae	Blue flag, Snake lily	eyewash	root		[18]
			powerful cathartic	rhizome		[24]
			root poultice used to treat sores	rhizome		[23]
					Rhizomes: iridals (17,26-dihydroxyiridal, 16-hydroxyiridal, 17-hydroxyiridal, 26-hydroxyiridal, 10-deoxy-17-hydroxyiridal, iriversical)	[247]
*Juglans nigra* L.	Jugland-aceae	Black walnut	bark infusion used on sores	bark		[15]
					Bark: juglone, α-hydroxyjuglone-4-glucoside, myricetin, myricitrin, sakuranetin, sakuranin, and neosakuranin	[248]
					Unripe fruit: naphthoquinones (dihydroplumbagin, 3-methylplumbagin, isoplumbagin)	[249]
					Husk: naphthoquinones (juglone, plumbagin, regiolone), sterols (stigmasterol, β-sitosterol), flavonoids (taxifolin, kaempferol, quercetin, myricetin)	[250]
					Leaf EO: α-Pinene (6.3–11.4%), β-caryophyllene (17.3–20.4%), germacrene D (7.1–22.5%), juglone (1.0–8.8%)	[251]
*Juncus effusus* L.	Juncaceae	Common rush	plant decoction used as emetic	plant		[15]
					Medullae: *p*-Coumaroyl glycerides (juncusyl esters A and B)	[252]
					Plant: cinnamoylglycerols (1-*O*-coumaroylglycerol, 1-*O*-feruloylglycerol, 1-*O*-coumaroylglycerol, juncusyl ester A, 1-*O*-(4-methoxycinnamoyl)glycerol, 1-*O*-(4-methoxycinnamoyl)-2,3-*O*-isoppropylidene-sn-glycerol, 2-*O*-coumaroylglycerol, 2-*O*-(4-methoxycinnamoyl)glycerol, 1,2-di-*O*-feruloylglycerol, 1,3-di-*p*-coumaroylglycerol)	[253]
					Plant: 8-dihydroxy-1,7-dimethyl-6-vinyl-10,11-dihydro-dibenz[*b,f*] oxepin (showed brine shrimp lethality)	[254]
					Stems: cycloartane glucosides (juncosides II–V)	[255]
					Plant: cycloartane triterpenoids (lagerenol, cycloartane-3β,24,25-triol, cycloart-22Z-ene-3β,25-diol, sterculin A, cycloart-25-ene-3β,24-diol, 3-hydroxycycloart-25-ene-24-one, 24,25-epoxycycloartan-3β-ol)	[256]
					Plant: cycloartane glucoside juncoside I	[257]
					Medullae: phenanthrenes (junceunins E–G, dehydrojuncuenins D–E); junceunin E cytotoxic to MCF-7 and HeLa cells	[258]
					Underground parts: phenanthrenes (dehydroeffusol, juncusol); compounds showed UVA light-enhanced antimicrobial activities due to DNA binding	[259]
					Plant: phenanthrenes (4-ethenyl-9,10-dihydro-1,8-dimethyl-2,7-phenanthrenediol, 4-ethenyl-9,10-dihydro-7-methoxy-1,8-dimethyl-2-phenanthrenol, 4-ethenyl-9,10-dihydro-3,8-dimethyl-1,7-phenanthrenediol, 4-ethenyl-9,10-dihydro-7-methoxy-3,8-dimethyl-1-phenanthrenol, 4-ethenyl-9,10-dihydro-7-hydroxy-8-methyl-2-phenanthrenecarboxylic acid)	[260]
					Plant: phenanthrenes (junceunin F 2-methyl ether, 4-formyl-9,10-dihydro-3,7-dihydroxy-2,8-dimethylphenanthrene, 5-ethenyl-9,10-dihydro-1,7-dimethyl-2,3-phenanthrenediol, 9,10-dihydro-1,7-dihydroxy-4-(1-hydroxyethyl)-2,8-dimethylphenanthrene, 9,10-dihydro-6,6-dihydroxy-5-(1-hydroxyethyl)-1,7-dimethylphenanthrene, 9,10-dihydro-2,6-dihydroxy-5-(1-methoxyethyl)-1,7-dimethylphenanthrene, 4-ethenyl-9,10-dihydro-7-hydroxy-8-methyl-1-phenanthrenecarboxylic acid)	[261]
					Plant: phenanthrenes (2-hydroxy-7-(hydroxymethyl)-1-methyl-5-vinyl-9,10-dihydrophenanthrene, 2-hydroxy-6-(hydroxymethyl)-1-methyl-5-vinyl-9,10-dihydrophenanthrene, 2-hydroxy-5-(hydroxymethyl)-1,7-dimethyl-9,10-dihydrophenanthrene, 2,7-dihydroxy-5-(hydroxymethyl)-1,8-dimethyl-9,10-dihydrophenanthrene, 2-hydroxy-5-(hydroxymethyl)-7-methoxy-1,8-dimethyl-9,10-dihydrophenanthrene, 5-(1-ethoxy)-2,7-dihydroxy-1,8-dimethyl-9,10-dihydrophenanthrene, 2-hydroxy-1,7-dimethyl-9,10-dihydrophenanthro-[5,6-b]-4′,5′-dihydro-4′,5′-dihydroxyfuran)	[262]
					Plant: phenanthrene glucosides (Effusides I–V)	[263]
					Aerial parts: phenanthrenes (7-carboxy-2-hydroxy-1-methyl-5-vinyl-phenanthrene, 2,7-dihydroxy-1-methyl-5-aldehyde-9,10-dihydrophenanthrene, dehydroeffusol, dehydrojuncusol, 7-carboxy-2-hydroxy-1-methyl-5-vinyl-9,10-dihydrophenanthrene, 8-carboxy-2-hydroxy-1-methyl-5-vinyl-9,10-dihydrophenanthrene, effusol, and juncusol; effusol and juncusol showed anxiolytic and sedative activities)	[264]
					Medullae: diterpenoid effusenone A, phenanthrene 5-(hydroxymethyl)-1-methylphenanthrene-2,7-diol, pyrenes 1-methylpyrene-2,7-diol and 7-methoxy-8-methylpyren-2-ol	[265]
					Medullae: phenanthrenes (effusol, dehydroeffusol, dehydroeffusal)	[266]
					Medullae: phenanthrenes (effusol, dehydroeffusol, juncusol, dehydrojuncusol, juncuenin B, dehydrojuncuenin B, juncuenin D, and effususol A), flavonoids (luteolin and luteolin 5-methyl ether), and 4-hydroxy-2,3-dimethyl-2-nonen-4-olide	[267]
					Plant: tetrahydropyrene glucosides (4,5,9,10-tetrahydro-2,7-dihydroxy-1,6-dimethylpyrene monoglucoside and diglucoside)	[268]
					Medullae: phenanthrene dimers (effususins A–D); effususins A and B showed cytotoxic activity against several tumor cell lines; effususin B showed inflammatory activity (inhibition of NO production in LPS-stimulated RAW 264.7 cells)	[269]
					Phenanthrenes from medullae of *Juncus effusus* show cytotoxic activity against several tumor cell lines; some also show inhibition of NO production indicating anti-inflammatory potential	[270]
					The phenanthrene dehydroeffusol shows anxiolytic and sedative effects (mouse model)	[271]
					The phenanthrenes effusol and dehydroeffusol activate GABA_A_ receptors, explaining the traditional Chinese use of the plant as a sedative and anxiolytic agent	[272]
*Juniperus virginiana* L.	Cupress-aceae	Eastern red cedar	decoction of berries given for worms; infusion of some part taken for colds; ointment used on skin diseases	various		[15]
					Bark EO: α-pinene (77.5%)	[273]
					Leaf EO: α-pinene (2.3–6.5%), sabinene (2.8–8.7%), limonene (4.1–5.0%), safrole (18.8–22.3%), methyl eugenol (11.9–13.8%), elemol (10.6–13.6%), elemicin (6.8–7.1%)	[273]
					Berry EO: limonene (63.1%), elemol (18.4%)	[273]
					Wood EO: α-cedrene (27.2–35.0%), β-cedrene (7.7%), thujopsene (27.6–30.0%), cuparene (2.0–6.3%), cedrol (4.0–15.8%), widdrol (1.0–2.0%)	[274]
					Wood EO: α-cedrene (4.0%), β-cedrene (2.0%), thujopsene (30.1%), cedrol (38.8%), widdrol (5.6%)	[275]
					Wood EO: α-cedrene (41.4%), β-cedrene (7.5%), *cis*-thujopsene (20.0%), cedrol (13.4%)	dT ^b^
					Leaves: podophyllotoxin	[276]
*Lactuca canadensis* L.	Asteraceae	Canada lettuce	infusion taken for pain and calming nerves	plant		[15]
					Roots: sesquiterpene lactones (3-epizaluzanin C glucoside, 9-hydroxydehydroleucodin glucoside, zaluzanin C, 11β,13-dihydrozaluzanin C, 3-epizaluzanin C, 11β,13-dihydro 3-epizaluzanin C, vernoflexuoside, 11β,13-dihydro vernoflexuoside, macrocliniside A, ixerin F, picriside B, santamarin, 11β,13-dihydro santamarin, armexifolin, 1-epidehydroisoerivanin, armefolin, 1-epiisoerivanin, 3α-hydroxyreynosin and 1-epierivanin)	[277]
*Liatris spicata* (L.) Willd.	Asteraceae	Blazing star	tonic, tincture used on pains	root		[15]
					Flavonoid glycosides: quercetin 3-glucoside, quercetin 3-rutinoside, and quercetin 3-glucoside-7-rhamnoside	[278]
					Leaf: major volatiles: α-pinene, mesityl oxide, β-pinene, myrcene, 2,4-heptadienal, β-caryophyllene, germacrene D, caryophyllene oxide	[279]
					Aerial parts: guaianolide sesquiterpenoid spicatin	[280]
					Corms (underground stems): sterols (stigmasterol and its 3-*O*-glucoside), triterpene (obtusifoliyl acetate), benzofurans: (euparin and 6-hydroxy-3-methoxytremetone), phenolic acids (protocatechuic, vanillic and ferulic acid) and a sesquiterpene lactone igalan. Iglan showed cytotoxic activity on Hep-G2 cells	[281]
*Lindera benzoin* (L.) Blume	Lauraceae	Spicebush	infusion taken for measles, cough	bark		[15]
			infusion of leaves taken for coughs, colds, flu	leaves		[22]
					Leaf EO: 6-methyl-5-hepten-2-one (42.9%), β-caryophyllene (7.7%), bicyclogermacrene (5.1%), δ-cadinene (4.9%), and (*E*)-nerolidol (4.8%)	[282]
					Twigs EO: α-pinene (5.9%), sabinene (6.8%), α-phellandrene (4.2%), 1,8-cineole (45.4%), α-terpineol (6.8%)	[283]
					Fruit EO: myrcene (4.7%), α-phellandrene (64.6%), β-phellandrene (11.2%)	[283]
					Fruit: (6*Z*,9*Z*)-pentadecadien-2-one, (6*Z*,9*Z*,12*Z*)-pentadecatrien-2-one, (*Z*)-nerolidol, isolinderanolide, isolinderenolide, isoobtusilactone A, obtusilactone A, isoobtusilactone, obtusilactone, and linderanolide	[284]
*Liquidambar styraciflua* L.	Altingiaceae	Sweet gum	inner bark for diarrhea, externally for wounds, sores, ulcers	bark		[15]
					Bark: shikimic acid	[285]
					Bark: pentacyclic triterpenoids (25-acetoxy-3α-hydroxyolean-12-en-28-oic acid, 3α,25-dihydroxyolean-12-en-28-oic acid, 6β-hydroxy-3-oxolup-20(29)-en-28-oic acid, and 3,11-dioxoolean-12-en-28-oic acid); 25-acetoxy-3α-hydroxyolean-12-en-28-oic acid showed broad cytotoxic activity against a panel of human tumor cell lines	[286]
					Bark: polyphenolics (shikimic acid, gallic acid, vanillic acid)	[287]
					Cones: pentacyclic triterpenoids (6β,30-dihydroxy-3-oxolup-20(29)-en-28-oic acid, 3α-hydroxy-11-oxoolean-12-en-28-oic acid, and massagenic acid G)	[288]
					Leaves: polyphenolics (gallic acid, isorugosin, casuarictin, quercetin-3-*O*-glucoside, myricetrin, quercetin, myricetin); extract showed hepatoprotective activity	[289]
					Aerial parts: β-sitosterol, lupeol, oleanolic acid, ursolic acid, luteolin, orientin, isoorientin, kaempferol 3-*O*-α-rhamnoside, and kaempferol 3-*O*-β-glucoside. Extract showed acetylcholinesterase inhibitory activity	[290]
					Leaf EO: α-Pinene (26.2–28.0%), β-pinene (10.1–11.3%), Limonene (20.7–22.3%)	[291]
					Stem EO: α-Pinene (11.1–16.0%), β-pinene (4.4–8.6%), Limonene (11.2–12.9%), β-caryophyllene (5.4–6.9%), germacrene D (6.7–10.9%)	[291]
*Liriodendron tulipifera* L.	Magnoli-aceae	Tulip tree	bark infusion taken for pinworms, cholera, coughs, rheumatism	bark		[15]
					Bark: lignans (lirionol, syringic acid methyl ester, pinoresinol, syringaresinol), aporphine alkaloids (*O*-methyl-*N*-noraporphine, *N*-(2-hydroxy-2-phenylethyl)-benzamide)	[292]
					Bark: aporphine alkaloids (asimilobine, norushinsunine, norglacine, liriodenine, anonaine, oxoglaucine); the aporphine alkaloids showed antiplasmodial activity	[293]
					Leaves: germacranolide sesquiterpenoids (peroxyferolide, lipiferolide); showed antiplasmodial and cytotoxic activities	[293]
					Leaves: aporphine alkaloids (anonaine, norstephalagine, liridinine, nornuciferine, caaverine, lirinidine, lysicamine), a coumarin (scopoletin), a germacranolide (epitulipinolide diepoxide), polyphenolics (β-orcinol carboxylate, syringaldehyde, syringic acid, vanillic acid), sterols (β-sitosterol, stigmasterol); anonaine, liridinine, lysicamine, and epitulipinolide diepoxide significantly inhibited prolifertion of A375 melanoma cells	[294]
					Leaves: germacranolide (dihydrochrysanolide, 11,13-dehydrolanuginolide, laurenbiolide) and guaianolide (β-cyclolipiferolide) sesquiterpenoids	[295]
					Aerial parts: lignans (sesamin, syringaresinol, dihydrodehydrodiconiferyl alcohol, salvinal, guaicylglycerol-8-*O*-4′-dihydroconiferyl ether, guaiacylglycerol-8-*O*-4′-sinapyl alcohol ether, tanegool, 5,5′-dimethoxy-7-oxolariciresinol), phenolics (3-hydroxy-4-methoxyacetophenone, 4-acetoxymethylphenol), germacranolide (paramicholide), and blumenol A	[296]
					Roots: germacranolides (tulipinolide, epitulipinolide)	[297]
					Leaf EO: (*Z*)-β-Ocimene (6.1–59.4%), (*E*)-β-ocimene (4.4–24.0%), β-elemene (8.2–23.5%), germacrene D (4.8–43.5%), bicyclogermacrene (3.0–21.5%); β-ocimenes cytotoxic to MDA-MB-231 and Hs578T cells	[298]
					Bark EO: α-Pinene (6.7–11.3%), camphene (1.1–5.0%), β-pinene (6.9–19.1%), myrcene (2.4–11.7%), limonene (4.5–12.0%), β-phellandrene (up to 13.7%), (*Z*)-β-ocimene (30.6–53.9%), bornyl acetate (2.6–13.3%)	[299]
*Lobelia cardinalis* L.	Campanu-laceae	Cardinal flower	root infusion for worms, rheumatism; leaf infusion for colds, fever; root poultice for sores	root, leaves		[15]
					Aerial parts: alkaloid lobinaline	[300]
					Hairy root culture: diacetylene triol lobetyol + glucosides lobetyolin and lobetyolinin	[301]
					Leaves: anthocyanin cyanidin-3-*O*-[6-*O*-(4-*O*-*E*-*p*-coumaroyl-*O*-α-rhamnopyranosyl)-β-glucopyrano]-5-*O*-β-glucopyranoside	[302]
*Lobelia inflata* L.	Campanu-laceae	Indian tobacco	root poultice used on pains; root/leaf poultice used on ringworm, insect bites	root, leaves		[15]
					Hairy root culture: diacetylene triol lobetyol + glucosides lobetyolin and lobetyolinin	[303]
					Aerial parts: piperidine alkaloids (lobeline, lobelanine, norlobeline, norlobelanine, lobelanidine, norallosedamine, 8-ethyl-10-phenylnorlobelionol, 8-ethyl-10-phenyllobelionol)	[304]
					Aerial parts: piperidine alkaloids (8,10-diethyllobelidione, 8,10-diethyllobelidione, 8-ethyl-10-phenyl-norlobelionol, 8-ethyl-10-phenyl-dehydrolobelionol, 8-ethyl-10-phenyl-dehydrolobelionol, lobeline, lobelidine, lobelanine)	[305]
*Lobelia siphilitica* L.	Campanu-laceae	Great blue lobelia	root infusion for worms; leaf infusion for colds, fever	root, leaves		[15]
					Aerial parts: piperidine alkaloids (lobeline, cis-8,10-diphenyllobelidiol, (*S*)-2-[(2*S*,6*R*)-1-methyl-6-(2-oxo-2-phenylethyl)piperidin-2-yl]-1-phenylethyl acetate, 6-[(*E*)-2-(3-methoxyphenyl)ethenyl]-2,3,4,5-tetrahydropyridine) and the diacetylene lobetyolin	[306]
*Lycopus virginicus* L.	Lamiaceae	Virginia bugleweed	tea; root applied to snakebite	plant, root		[15]
					Aerial parts: flavone glucuronides (7-*O*-β-d-glucuronides of apigenin, acacetin, and luteolin as well as the methyl ester of apigenin 7-*O*-β-d-glucuronide)	[307]
*Magnolia acuminata* (L.)	Magnoli-aceae	Cucumber magnolia	bark infusion for toothache	bark		[15]
					Root bark: lignans (calopiptin, galgravin, veraguensin, and acuminatin)	[308]
					Root bark: alkaloids (anolobine, *N*-methyllidcarpine methiodide, *N*,*N*′-dimethyl-2,11,-dihydro-1,10-dimethoxyaporphine iodide), lignans (calopiptin, galgravin, veraguensin, acuminatin), sesquiterpene lactone (costunolide), sterol (β-sitosterol)	[309]
					Leaves: alkaloids (asimilobine, liriodenine, norarmepavine, roemerine, armepavine, magnocurarine, magnoflorine)	[310]
*Menispermum canadense* L.	Menisperm-aceae	Common moonseed	root used for skin diseases	root		[15]
					Roots: alkaloid dauricine	[311]
					Aerial parts: alkaloid acutumine	[312]
					Roots: alkaloids (acutumine, acutumidine, dauricine, daurinoline, *N*′-desmethyldauricine, magnoflorine, *N*,*N*-dimethyllindcarpine, dehydrocheilanthifoline)	[312]
*Monarda didyma* L.	Lamiaceae	Scarlet beebalm	infusion abortifacient; poultice for colds, headache	leaves	Several essential oil chemotypes are known	[15]
					Floral EO: sabinene (5.0%), γ-terpinene (5.3%), *p*-cymene (11.0%), linalool (64.5%)	[313]
					Leaf EO: linalool (74.2%), bornyl acetate (5.7%), germacrene D (5.3%)	[313]
					Commercial EO (Pam′innov, Le Chaffaut-Saint-Jurson, Provence, France): geraniol (89.5%)	[314]
					Leaf EO: δ-3-carene (4.5%), *p*-cymene (10.5%), γ-terpinene (9.3%), thymol (57.3%); EO showed antifungal and DPPH radical inhibitory activities	[315]
					Leaf EO: γ-terpinene (7.0%), α-terpinene (7.0%), *p*-cymene (20.1%), borneol (11.7%), 1-octen-3-ol (21.7%), thymol (12.3%), thymoquinone (10.1%)	[316]
					Leaf EO: γ-terpinene (6.6%), *p*-cymene (33.9%), thymol (38.0%), thymoquinone (12.8%)	[316]
					Leaf EO: *p*-cymene (17.0%), carvacrol (69.7%)	[316]
					Leaf EO: *p*-cymene (17.0%), linalool (29.3%), 1-octen-3-ol (9.8%), thymol (5.5%), thymoquinone (22.3%)	[316]
					Leaf EO: *p*-cymene (21.2%), 1-octen-3-ol (7.1%), carvacrol (46.8%), thymoquinone (21.3%)	[316]
					Aerial parts EO (*M. didyma* var 80-1A): *p*-cymene (8.2%), linalool (55.4%), geraniol (20.7%); EO inhibited mycelial growth spore germination of *Botrytis cinerea*	[317]
					Aerial parts EO: *p*-cymene (12.6%), γ-terpinene (15.9%), thymol (41.2%), carvacrol (15.2%); EO inhibited mycelial growth spore germination of *Botrytis cinerea*	[317]
					Aerial parts EO: δ-3-carene (4.1–4.5%), *p*-cymene (10.2–10.3%), γ-terpinolene (9.2%), thymol (59.4–64.3%); EO showed anticandidal and antibacterial activity.	[318]
					Aerial parts EO: *p*-cymene (10.3%), terpinolene (9.2%), thymol (59.3%); EO showed anti-germination activity against several ′′weed′′ seeds	[319]
					Leaves and flowers: flavonoids (rutin, hyperoside, quercitrin, luteolin, quercetin)	[320]
*Monarda fistulosa* L.	Lamiaceae	Wild bergamot	fevers, colds	plant	Several subspecies are known	[15]
					Aerial parts EO: geraniol (86.8%)	[321]
					Leaf EO: *p*-cymene (9.2%), thymol (72.9%), carvacrol (6.8%), thymoquinone (5.9%)	[316]
					Aerial parts EO: myrcene (8.1%), α-phellandrene (13.7%), β-phellandrene (17.0%), *p*-cymene (13.5%), thymol (26.5%)	[322]
					Aerial parts EO: *p*-cymene (35.4%), 1-octen-3-ol (10.3%), carvacrol (39.1%); the EO and carvacrol showed good mosquito (*Aedes aegypti*) repellent activity	[323]
					Aerial parts EO: myrcene (8.6–8.7%), α-phellandrene (13.7–14.0%), *p*-cymene (13.2–13.3%), thymol (28.4–33.4%); EO showed anticandidal and antibacterial activity	[318]
					Leaf EO: α-terpineol (35.9%, 99% l-enantiomer), thymol methyl ether (14.0%), linalool (5.0%, 100% l-enantiomer)	WNS ^c^
*Oenothera biennis* L.	Onagraceae	Evening primrose	eye conditions due to asthma, allergies; poultice on boils	root		[15]
			poultice used on hemorrhoids	leaves		[21]
					Roots: oenotheralanosterol A, oenotheralanosterol B	[324]
					Roots: oenotheralanosterol A, oenotheralanosterol B	[325]
					Roots: gallic acid (antifungal)	[326]
					Roots: 6-(13,14-ciacetyloxyprenyl)-1,3,7-trimethoxyxanthone, eicos-9-enoyl-α-d-glucopyranosyl-(6→1′′)-α-d-glucopyranoside	[327]
					Roots: oleanolic acid, maslinic acid, β-sitolsterol, gallic acid, 2,7,8-trimethylellagic acid, tetramethylellagic acid, 2-methyl-7-oxotritetracont-1,5,dien-21ol, 18-hydroxypentacos-21-enoic acid, 5-methyl-27-oxotriacont-4-en-24-ol, and 3,5-dihydroxy-4-pent-4′-enoyl-1′-oxymethylbenzoic acid	[328]
					Seed oil: linoleic acid, sterols (campesterol, β-sitosterol, Δ^5^-avenasterol)	[329]
					Seeds: catechin, epicatechin, gallic acid	[330]
					Seeds: protocatechuic acid	[331]
					Aerial parts: phenolics (galloylglucose, gallic acid, oenothein B, quercetin 3-*O*-glucuronide, kaempferol 3-*O*-glucuronide)	[332]
*Panax quinquefolius* L.	Araliaceae	American ginseng	root used as tonic	root		[15]
					Root (wild): ginsenosides [Rb_1_ (2.81%), Rb_2_ (0.09%), Rc (0.42%), Rd (0.29%), Re (1.42%), and Rg_1_ (0.94%)]	[333]
					Root: ginsenosides (Rb_1_, Rb_2_, Rc, Rd, Re, Rf and Rg_1_)	[334]
					Root (cultivated): ginsenosides [Rb_1_ (3.70%), Rb_2_ (0.05%), Rc (0.41%), Rd (0.42%), Re (0.50%), and Rg_1_ (0.13%)]	[335]
					Root (cultivated): ginsenosides [Rb_1_ (1.85%), Rb_2_ (0.04%), Rb_3_ (0.04%), Rc (0.29%), Rd (0.29%), Re (2.05%), Rg_1_ (0.25%), and F_11_ (0.20%)]	[336]
					Root (cultivated): polyacetylenes (falcarinol, panaxydol)	[337]
					Root (cultivated): ginsenosides [Rb_1_ (4.94%), Rb_2_ (0.04%), Rc (0.39%), Rd (0.60%), Re (1.75%), and Rg_1_ (0.13%)]	[338]
					Leaves (wild): ginsenosides [Rb_1_ (0.17%), Rb_2_ (1.04%), Rc (0.18%), Rd (1.08%), Re (0.93%), and Rg_1_ (0.14%)]	[333]
					Leaves (cultivated): ginsenosides [Rb_1_ (0.28%), Rb_2_ (1.82%), Rb_3_ (4.64%), Rc (0.56%), Rd (2.82%), Re (3.42%), Rg_1_ (0.96%), and F_11_ (1.94%)]	[336]
					Review of chemical analysis of *P. quinquefolius*	[339]
					Review of pharmacology and toxicology of *P. quinquefolius*	[340]
					Review of ginsenosides in *P. quinquefolius*	[341]
					Review of pharmacology of *P. quinquefolius*	[342]
*Panax trifolius* L.	Araliaceae	Dwarf ginseng	root used as tonic	root		[22]
					Leaves: flavonoids (kaempferol-3,7-dirhamnoside and kaempferol-3-gluco-7-rhamnoside), ginsenosides (ginsenoside-Rd, -Rc, -Rb3 and notoginsenoside-Fe)	[343]
					Leaves: ginsenosides (Ro, Rb1, Rb2, Rc)	[344]
*Parthenocissus quinquefolia* (L.) Planch.	Vitaceae	Virginia creeper	infusion taken for jaundice			[15]
					Stem: resveratrol oligomers, parthenocissins A and B, were isolated in addition to three known stilbenes (resveratrol, piceatannol, resveratrol 3-glucoside)	[345]
					Stem: oligostilbenes, parthenocissins M and N, together with two known compounds, miyabenol C and ϵ-viniferin	[346]
					Leaves: β-amyryl palmitate; shows thrombin inhibitory activity	[347]
*Passiflora incarnata* L.	Passiflor- aceae	Passion flower	root infusion used for boils, earache, to wean babies; poultice for wounds	root		[15]
					Plant: *C*-Glycosidic flavonoids (schaftoside, isoschaftoside, isovetexin-2″-*O*-glucopyranoside and isoorientin-2″-*O*-glucopyranoside)	[348]
					Plant: flavonoid glycosides (vicenin-2. schaftoside, isoschaftoside isoorientin-2′′-*O*-glucoside, isoorientin, isovitexin-2′′-*O*-glucoside, swertisin, orientin isovitexin, vitexin	[349]
					Plant: flavonoid glycoside (isoscoparin-2′′-*O*-glucoside)	[350]
					Plant: *C*-glycosidic flavonoid (6-β-D-glucopyranosyl-8-β-d-ribopyranosyl apigenin)	[351]
					The phytochemistry of *P. incarnata* has been reviewed	[352]
*Phytolacca americana* L.	Phytolac-caceae	Pokeweed	poultice used for ulcers; root infusion used for eczema	root		[15]
					Roots: triterpenoid saponins (phytolaccosides A, D, E)	[353]
					Roots: triterpenoid saponin (phytolaccoside B)	[354]
					Roots: triterpenoid saponins (phytolaccasaponins B, E, G)	[355]
					Roots: triterpenoid saponins (phytolaccasaponins N1–N5; esculentoside H, esculentoside A = phytolaccoside E, esculentoside M, esculentoside B = phytolaccoside B, esculentoside S, esculentoside R-28-*O*-glucoside, esculentoside L)	[356]
					Roots: phytosterol α-spinasterol	[357]
*Pinus virginiana* Mill.	Pinaceae	Pine	wash for skin ulcers/sores; sap used on stubborn sores; syrup from inner bark for coughs/congestion; inner bark used for intestinal worms and parasites.	bark		[18]
					Bark EO: α-pinene (43.1%), β-pinene (24.8%), β-phellandrene (13.9%)	[273]
					Leaf EO: α-pinene (22.8%), β-pinene (25.1%), β-phellandrene (14.3%), α-terpineol (8.7%)	[273]
*Plantago lanceolata* L. ^a^	Plantagin-aceae	Narrowleaf plantain	infusion or poultice used for bites and stings	plant		[15]
					Herb: purpureaside A, lavandulifolioside B, acteoside, luteolin-3′,7-diglucuronide, isoacteoside, luteolin-7-glucuronide, and luteolin	[358]
					Herb: phenolic acids: *p*-hydroxybenzoic acid, vanillic acid, gallic acid, cinnamic acid, chlorogenic acid (major); flavonoids: apigenin, luteolin, luteolin-7-*O*-glucoside. Extract shows antioxidant, COX-1-inhibitory, 12-LOX-inhibitory, and weak cytotoxic activity	[359]
					Herb: iridoid glycosides: aucubin and catapol	[360]
					Herb: iridoid glycosides: aucubin and catapol	[361]
					Herb: acteoside, aucubin, catalpol	[362]
					Herb: acteoside, aucubin, catalpol	[363]
*Plantago major* L. ^a^	Plantagin-aceae	Common plantain	infusion or poultice used for bites and stings	plant		[15]
					Review, Herb: aucubin, melittoside, asperuloside, melampyroside, plantarenaloside, ixoroside, majoroside, 10-hydroxymajoroside, 10-acetoxymajoroside, acteoside, plantamajoside	[364]
					Review, Herb: caffeic acid derivatives (caffeic acid, chlorogenic acid, plantamajoside, acteoside), flavonoids (apigenin 7-glucoside, baicalein, hispidulin, hispidulin 7-glucuronide, homoplantaginin, luteolin 7-glucoside, luteolin 7-diglucoside, luteolin 6-hydroxy-4′-methoxy-7-galactoside, nepetin 7-glucoside, plantaginin, scutellarein), iridoid glycosides (asperuloside, aucubin, catapol, gardoside, geniposidic acid, majoroside, 10-acetoxymajoroside, 10-hydroxymajoroside, melittoside), triterpenoids (oleanolic acid, ursolic acid, 18β-glycyrrhetinic acid). Bioactivities of extracts includes wound healing activity, anti-inflammatory, analgesic, antioxidant, weak antibiotic, immuno modulating and antiulcerogenic activity	[365]
					*P. major* compounds showed antiviral activity: caffeic acid on herpesvirus (HSV-1) and adenovirus (ADV-3); chlorogenic acid on ADV-11	[366]
					Herb: ursolic acid, oleanolic acid	[367]
					Herb: ursolic acid, oleanolic acid	[368]
					Herb: isomarynoside, 10-hydroxymajoroside, β-sitosterol, ursolic acid	[369]
					Herb: ursolic acid, oleanolic acid	[370]
					Herb: α-linolenic acid, ursolic acid, oleanolic acid; SFE extract showed COX-2 inhibitory activity	[371]
*Platanus occidentalis* L.	Platanaceae	American sycamore	infusion of inner bark for cough, measles, urinary infection	bark		[15]
					Bark: anti-MRSA flavonoids (kaempferol 3-*O*-α-l-(2′′,3′′-di-*E*-*p*-coumaroyl)rhamnoside, kaempferol 3-*O*-α-l-(2′′-E-p-coumaroyl-3′′-*Z*-*p*-coumaroyl)rhamnoside, kaempferol 3-*O*-α-l-(2′′-*Z*-*p*-coumaroyl-3′′-*E*-*p*-coumaroyl)rhamnoside, and kaempferol 3-*O*-α-l-(2′′,3′′-di-*Z*-*p*-coumaroyl)rhamnoside)	[372]
*Podophyllum peltatum* L.	Berberi-daceae	Mayapple	anthelmintic, sores	root		[15]
			warts	resin		[15]
					Roots: aryltetralin lignans (podophyllotoxin, picropodophyllotoxin, α-peltatin, β-peltatin, desoxypodophyllotoxin)	[373]
					Roots: aryltetralin lignans (podophyllotoxin, 4′-demethylpodophyllotoxin, α-peltatin, β-peltatin, desoxypodophyllotoxin, podophyllotoxone, isopicropodophyllone, 4′-demethyldesoxypodophyllotoxin, 4′-demethylpodophyllotoxone and 4′-demethylisopicropodophyllone	[374]
					Plants: aryltetralin lignans (podophyllotoxin 4-*O*-β-d-glucopyranoside, epipodophyllotoxin 4-*O*-β-d-glucopyranoside, 4¢-demethylpodophyllotoxin, α-peltatin, epipodophyllotoxin, podophyllotoxin, β-peltatin, 1,2,3,4-dehydrodesoxypodophyllotoxin)	[375]
*Polygala senega* L.	Polygalaceae	Seneca snakeroot	snakebite	root		[15]
					Root: triterpenoid saponin senegin-II	[376]
					Root: triterpenoid saponins (senegin III, senegin IV)	[377]
					Root: oligosaccharide esters (senegose A, senegose B, senegose C, senegose D, senegose E)	[378]
					Root: oligosaccharide esters (senegose F, senegose G, senegose H, senegose I)	[379]
					Root: oligosaccharide esters (senegose J, senegose K, senegose L, senegoseM, senegose N, senegose O)	[380]
					Root: triterpenoid saponins (senegin II, senegin III, *E*-senegasaponin A, *E*-senegasaponin B, *Z*-senegasaponin A, *Z*-senegasaponin B, *Z*-senegin II, *Z*-senegen III)	[381]
					Root: essential oil [hexanoic acid (33.6%), methyl salicylate (26.5%), n-hexanal (5.3%) and o-cresol (3.5%)]	[382]
					Root: triterpenoid saponins (senegin II, senegin III, senegin IV, senegasaponin A, senegasaponin B)	[383]
*Polygonum aviculare* L.	Polygon-aceae	Prostrate knotweed	fish poison	plant		[15]
					Plant: lignan aviculin; flavonoids (juglanin, avicularin, astragalin, and betmidin)	[384]
					Plant: naphthoquinone 6-methoxyplumbagin, also β-sitosterol, oleanolic acid, and 5,6,7,4′-tetramethoxyflavanone	[385]
					Aerial parts: flavonoids (avicularin, liquiritin, cinaroside)	[386]
					Plant: flavonol glucuronides [myricetin 3-*O*-β-d-glucuronide, mearsetin 3-*O*-β-d-glucuronide, quercetin 3-*O*-β-d-glucuronide, isorhamnetin 3-*O*-β-d-glucuronide, kaempferide 3-*O*-β-d-glucuronide, kaempferol 3-*O*-β-d-glucuronide, kaempferol 3-*O*-β-(2″-*O*-acetyl-β-d-glucuronide), isorhamnetin 3-*O*-β-(2″-*O*-acetyl-β-d-glucuronide), quercetin 3-*O*-β-(2″-*O*-acetyl-β-d-glucuronide), quercetin 3-*O*-β-(3″-*O*-acetyl-β-d-glucuronide), and kaempferol 3-*O*-β-(3″-*O*-acetyl-β-d-glucuronide)]	[387]
					Leaves: flavonoids (myricetin, quercetin, kaempferol, myricitrin, desmanthin-1, isoquercitrin, quercitrin, avicularin, juglanin) and gallic acid	[388]
					Aerial parts: flavonoids (avicularin, juglanin, myricitrin, isoastragalin, isoquercitrin, kaempferol-5,7-di-*O*-β-d-glucopyranoside, and kaempferol 5-*O*-α-l-rhamnopyranoside 5-*O*-β-d-glucopyranoside), lignan aviculin, and loliolide and 1,6-digalloylglucose	[389]
*Polygonum hydropiper* L.	Polygon-aceae	Marshpepper knotweed	fish poison	plant		[15]
					Plant: polygodial	[390]
					Plant: drimane sesquiterpenoids (warburanal, polygodial, isopolygodial, polygonal, isodrimeninol, drimenol, confertifolin)	[391]
					Plant: flavonoids [rutin (0.58–0.93%), hyperin (0.37–0.63%), isoquercitrin (0.08–0.38%), quercitrin (0.55–0.95%), catechin (0.06–0.09%), epicatechin (0.05–0.08%), quercetin (0.28–0.65%), kaempferol (0.28–0.53%), isorhamnetin (0.03–0.04%)]	[392]
					Leaves: drimane sesquiterpenoids (polygonic acid, 11-ethoxycinnamolide, polygodial acetal, valdiviolide, and fuegin), drimane norsesquiterpenoids (isopolygonal and polygonone)	[393]
					Leaves: flavonoids (7,4′-dimethylquercetin, 3′-methylquercetin, quercetin, isoquercitrin)	[394]
					Leaves: flavonoid sulfates (quercetin 3-sulfate, isorhamnetin 3,7-disulfate, and tamarixetin 3-glucoside-7-sulfate)	[395]
					Leaves: flavonoids [3-*O*-α-l-rhamnopyranosyloxy-3′,4′,5,7-tetrahydroxyflavone; 3-*O*-β-d-glucopyranosyloxy-4′,5,7-trihydroxyflavone; 6-hydroxyapigenin; 6″-*O*-(3,4,5-trihydroxybenzoyl) 3-*O*-β-d-glucopyranosyloxy-3′,4′,5,7-tetrahydroxyflavone; scutillarein; 6-hydroxyluteolin;3′,4′,5,6,7-pentahydroxyflavone; 6-hydroxyluteolin-7-*O*-β-d-glucopyranoside; quercetin 3-*O*-β-d-glucuronide; 2″-*O*-(3,4,5-trihydroxybenzoyl)quercitrin; quercetin)	[396]
					Sprout: drimane sesquiterpenoids (polygodial, warburganal)	[397]
					Sprout: flavonoid (2*R*,3*R*)-(+)-taxifolin (showed tyrosinase inhibition)	[398]
					Aerial parts: sucrose cinnamyl esters (hydropiperoside A, hydropiperoside B, vanicoside A, vanicoside B, vanicoside E)	[399]
					Sprout: essential oil [β-caryophyllene (9.3%), α-humulene (6.0%), (*E*)-β-farnesene (44.1%), (*E*)-nerolidol (6.9%), phytol (10.8%)]	[400]
					Leaves: essential oil (confertifolin, 22.9%)	[401]
*Polymnia canadensis* L.	Asteraceae	Whiteflower leafcup	Houma Native American use (not Cherokee) applied a leaf poultice to swellings	leaves		[15]
					Leaf EO: germacrene D (44.5–63.7%), β-caryophyllene (14.8–15.9%), α-humulene (3.9–5.1%)	WNS^c^
*Polymnia uvedalia* (L.) (syn. *Smallanthus uvedalia* (L.) Mack.)	Asteraceae	Leafcup, Bear′s foot	bruised root used on cuts, burns	root		[15]
					Germacranolide sesquiterpenoids (uvedalin, isouvedalin, 2′,3′-dehydromelnerin A, 9-hydroxy-2′,3′-dehydromelnerin A), *ent*-kaurane diterpenoids (*ent*-12-hydroxy-16-kauren-19-oic acid, *ent*-18-hydroxy-16-kauren-19-oic acid derivatives, *ent*-16-kauren-3,19-diol derivatives, *ent*-12,18-dihydroxy-16-kauren-19-oic acid derivatives	[247]
					Leaf EO: caryophyllane sesquiterpenoids: β-caryophyllene (16.5–24.5%), caryophyllene oxide (14.2–19.8%), caryophylla-4(12), 8(13)-dien-5β-ol (2.3–5.5%), 14-hydroxy-9-epi-(*Z*)-caryophyllene (4.3–8.2%), 14-hydroxy-9-epi-(*E*)-caryophyllene (6.2–8.9%)	WNS^c^
*Prunella vulgaris* L.	Lamiaceae	Heal-all	sore throat, cuts, burns	plant		[15]
					Leaf EO: selin-1 1-en-4α-ol (14.9%), *cis*-eudesma-6,11-diene (9.4%), 1,10-di-*epi*-cubenol (8.0%), spathulenol (5.8%) and germacrene D (5.1%)	[402]
					Leaf EO: aromadendrene (55.4%), cucumber alcohol (8.5%) and phytol (5.1%)	[403]
					Aerial parts: rosmarinic acid, ursolic acid, oleanolic acid	[404]
					Aerial parts: rosmarinic acid, ursolic acid, oleanolic acid	[405]
					Aerial parts: four triterpenes, i.e., betulinic acid, ursolic acid, 2α,3α-dihydroxyurs-12-en-28-oic acid, and 2α-hydroxyursolic acid	[406]
					Aerial parts: polyacetylenic acids (octadeca-9,11,13-triynoic acid and trans-octadec-13-ene-9,11-diynoic acid	[407]
					oleanane-skeleton triterpenoid saponins, 3β,4β,16α-17-carboxy-16,24-dihydroxy-28-norolean-12-en-3-yl 4-*O*-β-d-xylopyranosyl-β-d-glucopyranosiduronic acid, (3β,4β,16α)-17-carboxy-16,24-dihydroxy-28-norolean-12-en-3-yl β-d-glucopyranosiduronic acid methyl ester, and (3β,4β)-24-hydroxy-16-oxo-28-norolean-12-en-3-yl 4-*O*-β-d-xylopyranosyl-β-d-glucopyranosiduronic acid	[408]
					Aerial parts: 15 triterpene acids (oleanic acid, ursolic acid, 2α,3α,19α-trihydroxyurs-12-en-28-oic acid, 2α,3α-dihydroxyurs-12-en-28-oic acid, maslinic acid, 2α,3α,19α,23-tetrahydroxyurs-12-en-28-oic acid, 2α,3α,23-trihydroxyurs-12-en-28-oic acid, 2α,3β-dihydroxyurs-12-en-28-oic acid, 2α,3β,24-trihydroxyolea-12-en-28-oic acid, (12*R*,13*S*)-2α,3α,24,trihydroxy-12,13-cyclotaraxer-14-en-28-oic acid, 2α,3α,24-trihydroxyurs-12,20(30)-dien-28-oic acid, 2α,3α,24-trihydroxyolea-12-en-28-oic acid, 2α,3β,19α,24-tetrahydroxyurs-12-en-28-oic acid 28-*O*-d-glucopyranoside, 2α,3α,19α,24-tetrahydroxyurs-12-en-28-oic acid 28-*O*-d-glucopyranoside, prunvuloside A); four flavonoids (quercertin 3-*O*-β-d-glucopyranoside, kaempferol 3-*O*-α-l-rhamnopyranosyl(1→6)-β-d-glucopranoside, kaempferol 3-*O*-β-d-glucopyranoside, quercertin 3-*O*-α-l-rhamnopyranosyl(1→6)-β-d-glucopyranoside); four phenolics (caffeic acid, *p*-hydroxycinnamic acid, rosmarinic acid, and 2-hydroxy-3-(3′,4′-dihydroxyphenly)propanoic acid); and a diterpene (*trans*-phytol)	[409]
					Aerial parts: polyphenolics (butyl rosmarinate, ethyl rosmarinate, methyl rosmarinate, rosmarinic acid, 3,4,α-trihydroxy-methyl phenylpropionate, and *p*-coumaric acid)	[410]
					Aerial parts: phenolics (quercetin, rutin, rosmarinic acid, caffeic acid, chlorogenic acid ferulic acid, protocatechuic acid)	[411]
					Aerial parts: polygalacerebroside, ursolic acid, β-amyrin, quercetin, quercetin-3-*O*-β-d-galactoside, α-spinasterol, stigmasterol, β-sitosterol, daucosterol	[412]
*Prunus serotina* Ehrh.	Prunaceae	Black cherry	bark infusion for colds	bark		[15]
					Leaves: flavonoids (avicularin, noutrin, hyperoside, narcissin, rutin, quercetin 3-*O*-neohesperidoside, 3-*O*-(2″-*O*-α-l-rhamnopyranosyl)-β-d-galactopyranoside)	[413]
					Leaves: chlorogenic acid (1.08–2.30%), rutin (0.10–0.35%), hyperoside (1.20–2.23%), reynoutrin (0.26–0.44%), guajiverin (0.07–0.22%), avicularin (0.98–1.82%), juglanin (0.04–0.20%)	[414]
					Leaves: triterpenoids [corosolic acid (0.137%), olanolic acid (0.129%), ursolic acid (0.884%)]	[415]
					Leaves: hyperoside, prunin, ursolic acid	[416]
					Leaves: chlorogenic acid, hyperoside, benzaldehyde	[417]
					Leaf EO: benzyl alcohol (20.3%), benzaldehyde (12.1%), cinnamyl alcohol (4.7%), cinnamaldehyde (1.1%)	[416]
					Flowers: chlorogenic acid (0.63–1.90%), rutin (0.17–0.31%), hyperoside (0.80–1.59%), reynoutrin (0.08–0.21%), guajiverin (0.10–0.28%), avicularin (0.20–0.95%), juglanin (0.08–0.16%)	[414]
					Bark: triterpenoids (ursolic acid, ursolic aldehyde, 2α,3α-dihydroxyurs-12-en-28-oic acid)	[418]
					Bark: flavonoids (4′-methoxynaringenin, naringenin, dihydrokaempferol, eriodictyol)	[419]
*Pseudognaphalium obtusifolium* (L.) Hilliard and B.L. Burtt (syn. *Gnaphalium obtusifolium* L.)	Asteraceae	Rabbit tobacco	infusion of herb for coughs, colds, flu	herb		[22]
					Plant: flavonoid obtusifolin	[420]
					Plant: flavonoids (gnaphaliin A, methylgnaphaliin)	[421]
					Plant: flavonoid 3,5,7-trihydroxy-6,8-dimethoxyflavone	[422]
*Pycnanthemum flexuosum* (Walter) Britton, Sterns and Poggenb.	Lamiaceae	Mountain mint	leaf infusion for headache, colds, fevers	leaves		[15]
					Whole plant: vanillic acid 1-*O*-[(5-*O*-syringoyl)-β-Dapiofuranosyl]-(1→2)-β-d-glucopyranoside, (4*S*,5*R*)-4-hydroxy-5-phenyl-tetrahydrofuran-2-one, luteoline 7-*O*-[(6-*O*-acetyl)-β-d-allopyranosyl-(1→2)-β-d-glucopyranoside], 4′-*O*-methylhypolaetin 7-*O*-[6-*O*-acetyl-β-d-allopyranosyl-(1→2)-β-d-glucopyranoside], apigenin 7-*O*-[6-*O*-acetyl-β-d-allopyranosyl-(1→2)-β-d-glucopyranoside], isoscutellarein 4-*O*-methylether 7-*O*-[(6-*O*-acetyl)-β-d-allopyranosyl-(1→2)-β-d-glucopyranoside], apigenin 7-*O*-[6-*O*-(*p*-*E*-coumaroyl)-β-d-glucopyranoside], 3′-hydroxy-4-*O*-methylisoscutellarein 7-*O*-[(6-*O*-acetyl)-β-d-allopyranosyl-(1→2)-(6-*O*-acetyl)-β-d-glucopyranoside], acteoside, leucosceptoside A, martynoside, artselaeroside A, stachysoside B, and chlorogenic acid	[423]
*Quercus alba* L.	Fagaceae	White oak	bark infusion for dysentery, antiseptic, fever	bark		[15]
					Bark: tannins	[424]
*Ranunculus acris* L. ^a^	Ranuncu- laceae	Tall buttercup	leaf poultice for abcesses; leaf infusion for sore throat	leaves		[15]
					Aerial parts: ranunculin	[425]
*Rhamnus caroliniana* Walter	Rhamnaceae	Buckthorn	itching skin, sores	berries, bark		
					Bark: chrysophanol, physcion, ararobinol, orachrysone, 1-docosanol	[426]
					Bark EO: chrysarobin (24.2%), piperine (15.4%), and pacharin (7.5%)	[426]
*Rhus glabra* L.	Anacardi-aceae	Smooth sumac	bark decoction to wash blisters	bark		[15]
					Branches: methyl gallate, 3,5-dihydroxy-4-methoxybenzoic acid, gallic acid; methyl gallate and 3,5-dihydroxy-4-methoxybenzoic acid showed antibacterial activity	[427]
					Leaves: *myo*-inositol, 1-docosanol, β-sitosterol, β-sitosterol glucoside, mixture of homologous alkanes (C_14_–C_33_, major heptacosane)	[428]
*Rhus hirta* Harv. ex Engl.	Anacardi-aceae	Staghorn sumac	bark decoction to wash blisters	bark		[15]
					Fruits: major components: sumadin B-3-*O*-(2″-galloyl)-galactoside-3′′′-*O*-glucoside, 7-*O*-methyl-cyanidin-3-*O*-(2″-galloyl)-galactoside; shows anti-oxidant activity	[429]
					Fruits: major components: sumadin B-3-*O*-(2″-galloyl)-galactoside-3′′′-*O*-glucoside, 7-*O*-methyl-cyanidin-3-*O*-(2″-galloyl)-galactoside; shows anti-inflammatory activity	[430]
*Rhus* spp. (as above)	Anacardi-aceae		infusion of berries for urinary tract infections, thrush	berries		[22]
*Robinia pseudoacacia* L.	Fabaceae	Black locust	bark chewed as emetic	bark		[15]
					Bark: lectins (RPbAI and RPbAII)	[431]
					Bark: lectin RPbAI (xtal structure)	[432]
					Saplings: flavonoids (acacetin, secundiflorol I, mucronulatol, isomucronulatol, isovestitol)	[433]
					Leaves: flavonoid glycosides (7-*O*-β-d-glucuronopyranosyl-(1→2)[α-l-rhamnopyranosyl-(1→6)]-β-d-glucopyranosides of acacetin, apigenin, diosmetin, and luteolin)	[434]
					Roots: lectins (RPrAI and RPrAII)	[435]
*Rubus allegheniensis* Porter	Rosaceae	Allegheny blackberry	leaf infusion for diarrhea	leaves		[15]
					Leaf extract: triterpenoids (tormentic acid, euscaphic acid, myrianthic acid, ziyu glycoside II, sericic acid, and 19-hydroxy-2,3-secours-12-ene-2,3,28-trioic acid 3-methyl ester)	[436]
*Rubus idaeus* L. ^a^	Rosaceae	Red raspberry	leaf infusion for pain; root infusion cathartic	roots, leaves		[15]
					Leaf extract: quercetin glucuronide, quercetin-3-glucoside and quercetin glucosylrhamnoside (rutin)	[437]
					Leaf extract: triterpenoid glycosides (3β-(*O*-β-d-glucopyranosyl)-olean-12-ene-1α,2α,3β-triol, 28-(*O*-β-d-glucopyranosyl)-urs-12-ene-2α,3β,19α-trihydroxy-28-oic acid, and 3β-(*O*-β-d-glucopyranosyl)-olean-12-ene-1α,2α,3β-trihydroxy-28-oic acid)	[438]
					Leaf extracts: tannins (ellagic acids, ellagitannins, sanguiin H-6 and H-10, and the trimers lambertianin D and lambertianin C, as well as methyl gallate), phenolic acids (chlorogenic acid, *p*-coumaric, ferulic, protocatechuic, gentisic, caffeoyltartaric, feruloyltartaric, and *p*-coumaroyl-glucoside acids, as well as *p*-hydroxybenzoic and vanillic acids), terpenoids (terpinolene, 3-oxo-α-ionol, α- and β-amyrin, squalene and cycloartenol)	[439]
*Rudbeckia fulgida* Aiton	Asteraceae	Orange coneflower	root used for ear medicine	root		[15]
					Leaf EO: β-caryophyllene (10.0%), γ-muurolene (8.9%), germacrene D (30.1%), δ-cadinene (17.8%)	[440]
*Rudbeckia hirta* L.	Asteraceae	Black-eyed Susan	root infusion taked for sexually transmitted diseases (STDs)	root		[15]
					Leaf EO: (2*E*)-hexenal (20.2%), (*E*)-β-ocimene (15.2%), γ-muurolene (8.1%), germacrene D (23.6%), δ-cadinene (16.2%)	[440]
*Rudbeckia laciniata* L.	Asteraceae	Souchan, Green-headed coneflower	tonic, skin wash	leaves		[15]
					Aerial parts: lignans ((+)-4,4′-*O*-diangeloylpinoresinol, (+)-4,4′-*O*-diangeloylmedioresinol, (+)-4,4′-*O*-diangeloylsyringaresinol, and (+)-syringaresinol)	[441]
					Aerial parts: flavonoid glycosides (quercetin 3-*O*-α-l-arabinofuranosyl-(1″→6″)-β-d-galactopyranoside, quercetin 3-*O*-α-l-arabinopyranosyl-(1″→6″)-β-d-galactopyranoside, quercetin-3-*O*-β-d-xylopyranosyl-(1″→2″)-β-d-glucopyranoside, and quercetin 3-*O*-β-d-glucopyranoside, isorhamnetin 3-*O*-β-d-glucopyranoside), quinic acid derivatives (3,5-*O*-*trans*-dicaffeoylquinic acid methyl ester, 3,5-*O*-*trans*-dicaffeoylquinic acid, 4,5-*O*-*trans*-dicaffeoylquinic acid methyl ester, 3,4-*O*-*trans*-caffeoylquinic acid methyl ester, 3,4-*O*-*trans*-caffeoylquinic acid, 5-*O*-*trans*-caffeoylquinic acid methyl ester, 3-*O*-*trans*-caffeoylquinic acid, and 3,5-*O*-*trans*-dicaffeoylepiquinic acid)	[442]
					Roots: sesquiterpene rudbeckianone	[443]
					Roots: sesquiterpene lactone rudbeckiolide	[444]
					Root extract: sesquiterpenoids (sesquithuriferol, igalan, lacinan-8-ol)	[445]
*Sambucus canadensis* L.	Adoxaceae	American elder	berry infusion for rheumatism; infusion of flowers taken for fever; leaves used to wash sores	plant		[15]
					Flowers: rutin	[446]
					Fruits: anthocyanins (cyanidin 3-sambubioside-5-glucoside, cyanidin 3,5-diglucoside, cyanidin 3-sambubioside, cyanidin 3-glucoside, cyanidin 3-*O*-(6-*O*-*Z*-*p*-coumaroyl-2-*O*-β-d-xylopyranosyl)-β-d-glucopyranoside-5-*O*-β-d-glucopyranoside, cyanidin 3-*O*-(6-*O*-*E*-*p*-coumaroyl-2-*O*-β-d-xylopyranosyl)-β-d-glucopyranoside-5-*O*-β-d-glucopyranoside (major), cyanidin 3-*O*-(6-*O*-*E*-*p*-coumaroyl-2-*O*-β-d-xylopyranosyl)-β-d-glucopyranoside)	[447]
					Fruits: anthocyanins [cyanidin 3-sambubioside-5-glucoside (0.0.11–0.19%), cyanidin 3,5-diglucoside (0.03–0.06%), cyanidin 3-sambubioside (0.03–0.04%), cyanidin 3-glucoside (0.04–0.06%), cyanidin 3-(*E*)-p-coumaroyl-sambubioside-5-glucoside (0.32–0.59%), cyanidin 3-p-coumaroyl-sambubioside (0.01–0.02%)]	[448]
					Fruits: anthocyanins (cyanidin 3-*O*-(6-*O*-*Z*-*p*-coumaroyl-2-*O*-β-d-xylopyranosyl)-β-d-glucopyranoside-5-*O*-β-d-glucopyranoside and cyanidin 3-*O*-(6-*O*-*E*-*p*-coumaroyl-2-*O*-β-d-xylopyranosyl)-β-d-glucopyranoside-5-*O*-β-d-glucopyranoside)	[449]
*Sanguinaria canadensis* L.	Papaver-aceae	Bloodroot	root decoction for cough	root		[15]
					Rhizome: alkaloids (sanguinarine, chelerythrine, protopine)	[450]
					Rhizome: alkaloids [protopine (0.32–0.74%), allocryptopine (0.34–0.77%), sanguinarine (1.38–4.45%), chelerythrine (0.99–2.57%), chelirubine (0.37–0.87%), chelilutine (0.78–1.83%), sanguilutine (0.49–1.03%)]	[451]
					Rhizome: alkaloids (sanguinarine and chelerythrine-antimycobacterial)	[452]
					Rhizome: alkaloids (sanguinarine, chelerythrine, protopine - anti-Helicobacter pylori)	[453]
					Rhizome: alkaloids [sanguinarine (2.81–3.96%), chelerythrine (1.38–2.08%)]	[454]
					Rhizome: alkaloids (sanguinarine, chelerythrine, sanguilutine, chelilutine, sanguirubine, chelirubine, protopine, and allocryptopine)	[455]
*Sassafras albidum* (Nutt.) Nees	Lauraceae	Sassafras	bark decoction for skin diseases, sexually-transmitted diseases; poultice for wounds and sores	bark		[17]
					Leaf EO: (3*Z*)-hexenol (2.5–9.9%), α-pinene (3.2–12.2%), camphene (0.3–5.4%), limonene (5.7–16.4%), linalool (3.5–6.7%), neral (9.9–18.1%), geranial (10.7–26.5%), β-caryophyllene (5.1–12.5%), caryophyllene oxide (0.4–19.0%)	[456]
					Root EO: safrole (85%), camphor (3.25%), and methyleugenol (1.10%)	[457]
					Bark EO: α-pinene (37.9–61.5%), camphene (2.9–5.1%), β-pinene (10.0–13.0%), 1,8-cineole (7.3–10.0%), camphor (1.7–4.6%), and α-terpineol (4.2–11.6%)	[458]
					Bark: sesamin, spinescin, β-sitosterol, hexatriacontanal, and 1-triacontanol; sesamin and spinescin showed antileishmanial activity	[459]
*Saururus cernuus* L.	Saururaceae	Lizard′s tail	mashed roots poultice for wounds	root		[15]
					Aerial parts: lignans (austrobailignan-5, veraguensin, guaiacin, saucernetin)	[460]
					Plant: lignans (manassantin A, manassantin B, saucerneol)	[461]
					Aerial parts: indole alkaloids (sauristolactam, cepharanone B)	[462]
					Aerial parts: lignans (saururin, saururenin, saururinone, austrobailignan 6, calopiptin, galbacin, zuonin A)	[463]
					Aerial parts: lignans (sauriol A, sauriol B)	[464]
					Aerial parts: lignans (licarin A, saucernetin, dihydroguaiaretic acid, sauriol A, sauriol B, saucerneol, and saucerneol methyl ether)	[465]
					Aerial parts: diterpenoid 12,13-dehydrogeranylgeraniol	[466]
					Aerial parts: lignans (manassantin B, 4-*O*-demethylmanassantin B)	[467]
					Stems and leaves: lignans (manassantin A, manassantin B, manassantin B_1_, 4-*O*-methylsaucerneol, verrucosin, austrobailignan-5)	[468]
*Scutellaria lateriflora* L.	Lamiaceae	Blue skullcap	root infusion for monthly period, diarrhea; root decoction to expel afterbirth; for breast pains, and for nerves	root		[15]
					Review	[469]
					Aerial parts EO: δ-cadinene (27%), calamenene (15.2%), β-elemene (9.2%), α-cubenene (4.2%), α-humulene (4.2%), and α-bergamotene (2.8%)	[470]
					Aerial parts: neo-clerodane diterpenoids (scutelaterin A, scutelaterin B, scutelaterin C, ajugapitin, and scutecyprol A)	[471]
					Herb: flavonoids baicalin and baicalein (aglycone)	[472]
					Aerial parts: indole alkaloids (melatonin, serotonin); flavonoids (baicalin, baicalein, wogonin, scutellarin)	[473]
					Herb: flavonoids (viscidulin III, chrysin, baicalein, oroxylin A, wogonin); phenolics (*trans*-verbascoside, *trans*-martynoside)	[474]
					Aerial parts: coumarins (scuteflorin A, scuteflorin B, decursin)	[475]
					Stem: flavonoids [scutellarin (0.08%)]; phenolic [acteoside (0.05%)]	[476]
					Root: flavonoids [baicalin (0.05%), baicalein (0.06%), wogonin (0.20%), oroxylin A (0.02%)]	[476]
					Leaf: flavonoids [scutellarin (0.92%), baicalin (0.05%)]	[476]
					Aerial parts: flavonoids (apigenin, luteolin, baicalein, wogonin, 6-methoxyluteolin 4′-methyl ether, isoscutellarin 8-*O*-β-d-glucuronide, apigenin 7-*O*-β-glucuronide, luteolin 7-*O*-β-glucuronide, baicalin, wogonin 7-*O*-β-glucuronide, wogonin 7-*O*-β-glucuronide methyl ester, eriodictyol, naringenin, naringenin 7-*O*-β-glucuronide); phenolics (acteoside, nonoside D, leucosceptoside A, martynoside, isoacteoside); lignan (syringaresinol 4′-*O*-β-d-glucopyranoside)	[477]
					Aerial parts: flavonoids (norwogonin-7-*O*-glucuronide, baicalin, dihydrobaicalin, galangin-7-*O*-glucuronide, dihydrooroxylin A-7-*O*-glucuronide, oroxylin A-7-*O*-glucuronide, wogonin-7-*O*-glucouronide, 5,7-dihydroxy-6,8-dimethoxyflavone-7-*O*-glucuronide, dihydrowogonin-7-*O*-glucuronide, baicalein, wogonin, oroxylin A, chrysin); phenolic (5-(β-d-glucosyloxy)-3-hydroxy-*trans*-stilbene-2-carboxylic acid)	[478]
*Senecio aureus* L.	Asteraceae	Golden ragwort	infusion of plant taken to prevent pregnancy/induce abortions	plant		[15]
					Eremophilane sesquiterpenoids (*trans*-9-oxofuranoeremophilane, 8α-ethoxy-10α*H*-eremophilenolide, 3α-angeloyloxy-9-oxo-10α*H*-furanoeremophilane)	[479]
*Silphium compositum* Michx.	Asteraceae	Rosin weed	tonic	plant		[15]
					Leaves: flavonoid glycosides (isorhamnetin 3-*O*-α-l-rhamnosyl (1′′′→6″)-*O*-β-d-galactopyranoside 7-*O*-β-l-apiofuranoside, quercetin 3-*O*-α-l-rhamnosyl(1′′′→6″)-*O*-β-d-galactopyranoside 7-*O*-β-l-apiofuranoside, quercetin 3-*O*-α-l-rhamnosyl (1′′′→6″)-*O*-β-d-galactopyranoside, quercetin 3-*O*-α-l-rhamnosyl (1′′′→6″)-*O*-β-d-glucopyranoside, isorhamnetin 3-*O*-α-l-rhamnosyl(1′′′→6″)-*O*-β-d-galactopyranoside, and quercetin 3-*O*-β-d-galactopyranoside)	[480]
*Solanum carolinense* L.	Solanaceae	Carolina horsenettle	leaf infusion for worms	leaves		[15]
					Leaves: steroidal glycoside (carolinoside) is shown to be *O*-(α-pentulopyranosyl)-(1→4)-*O*-(α-l-arabinopyranosyl)-(1→1)-d-glucopyranose	[481]
					Roots: ethyl *N*,*N*-bis(4-dimethylaminobutyl) carbamate (solaurethine). Other compounds reported for the first time in this species include solamine (principal base), cuscohygrine and anabasine	[482]
*Solidago odora* Aiton	Asteraceae	Goldenrod	bee stings, sore throat	flowers		[15]
*S. odora* Aiton fo. *odora*					Flowering parts EO: methyl chavicol (70.8%), myrcene (12.5%), methyl eugenol (5.8%), limonene (4.5%)	[483]
*S. odora* fo. *inodora* (A. Gray) Britton					Flowering parts EO: myrcene (31.3%), limonene (27.1%), (*E*)-methyl isoeugenol (12.9%), β-pinene (6.5%), α-pinene (5.4%), methyl eugenol (4.4%)	[483]
*Stillingia sylvatica* L.	Euphorbi-aceae	Queen′s delight	root tincture for STDs	root		[15]
					Roots: stillingia factors S_1_–S_6_ (2-hydroxydaphnetoxin diterpenoids)	[484]
*Symphyotrichum novae*-*angliae* (L.) G.L. Nesom (syn. *Aster novae*-*angliae* L.)	Asteraceae	New England aster	root poultice for pain	root		[15]
					Leaf EO: (2*E*)-hexenal (31.0%), α-pinene (16.4%), germacrene D (25.5%), δ-cadinene (14.3%)	[440]
*Thalictrum dioicum* L.	Ranuncu-laceae	Early meadowrue	root infusion for diarrhea	root		[15]
					Bis-benzylisoquinoline alkaloids (thalictropine, thalidoxine, pennsylvanine, thalmelatine, thalictrogamine	[485]
*Thalictrum dioicum*					Isopavine alkaloid thalidine	[486]
					Pallidine and corydine alkaloids	[487]
*Tilia americana* L.	Tiliaceae	American basswood	inner bark decoction for diarrhea, coughs, boils.	bark		[15]
*T. americana* var. *mexicana* (Schltdl.) Hardin					Flowers: quercetin and kaempferol derivatives; showed sedative and anxiolytic activity	[488]
*T. americana* var. *mexicana*					Flowers: tiliroside, quercetin, quercitrin, kaempherol; showed anxiolytic activity	[489]
*T. americana* var. *mexicana*					Flowers: quercetin; showed analgesic activity	[490]
*T. americana* var. *mexicana*					Flowers: quercetin, kaempferol; showed anxiolytic activity	[491]
*T. americana* var. *mexicana*					Flowers and leaves: flavonoids quercetin, rutin, isoquercetin); extract showed anticonvusant activity	[492]
*Tsuga canadensis* (L.) Carrière	Pinaceae	Eastern hemlock	bark poultice for itching skin; stem tips for kidneys	bark, leaves		[15]
					Foliar EO: α-pinene (17.6%), camphene (11.5%), isobornyl acetate (43.4%)	[493]
					Foliar EO: α-pinene (13.2%), camphene (7.8%), isobornyl acetate (42.9%)	[494]
					Foliar EO: tricyclene (1.6–5.1%), α-pinene (4.1–15.1%), camphene (3.0–11.1%), myrcene (0.5–21.1%), isobornyl acetate (22.0–55.8%), α-humulene (3.6–9.8%), germacrene D (1.4–21.3%)	[495]
					Foliar EO: tricyclene (3.1–7.8%), α-pinene (11.6–22.7%), camphene (7.8–15.9%), isobornyl acetate (32.8–50.7%), α-humulene (up to 9.2%), germacrene D (up to 6.4%)	[496]
					Foliar EO: α-pinene (13.9, 5.4%), camphene (13.3, 3.4%), limonene (6.0, 7.0%), piperitone (4.3, 7.7%), isobornyl acetate (38.6, 37.0%)	[497]
*Viburnum prunifolium* L.	Adoxaceae	Black haw shrub	bark infusion as tonic for female bleeding	bark		[15]
					Bark: biflavonoid amentoflavone	[498]
					Bark: iridoid glycosides (2-*O*-acetyldihydropenstemide, 2-*O*-*trans*-*p*-coumaroyldihydropenstemide, 2-*O*-acetylpatrinoside, and patrinoside)	[499]
					Bark: 1-methyl-2,3-dibutyl hemimellitate	[500]
*Vicia caroliniana* Walter	Fabaceae	Vetch	pains, rheumatism	plant		[15]
					Aerial parts EO: phytone (2.2–21.5%), methyl roughanate (1.9–29.5%), palmitic acid (9.9–28.1%), (*E*)-phytol (15.8–36.1%)	[501]
*Xanthorhiza simplicissima* Marshall	Ranuncu-laceae	Yellow root	root infusion for cramps, as tonic	root		[15]
					Root: alkaloids (berberine, jatrorrhizine, magnoflorine)	[502]
					Whole plant: alkaloids berberine and puntarenine	[503]
					Roots: bisbenzylisoquinoline alkaloids (obamegine and oxyacanthine)	[504]
*Zanthoxylum americanum* Mill.	Rutaceae	Common prickly ash	bark infusion for swollen joints	bark		[15]
					Bark: pyranocoumarins (dipetaline, alloxanthoxyletin, xanthoxyletin, xanthyletin) and lignans (sesamin, asarinin)	[505]
*Zanthoxylum clava*-*herculis* L.	Rutaceae	Hercules′s club	Houma tribe of Native Americans (not Cherokee) used the bark for toothache	bark		[15]
					Leaf EO: α-thujene (0.2–5.6%), limonene (43.6–73.0%), 1,8-cineole (12.9–43.3%), linalool (up to 11.3%)	[506]
					Bark EO: sabinene (47.0%), limonene (18.7%), terpinen-4-ol (12.9%)	[507]
					Bark: asarinin, sesamin, neoherculin, xanthoxylol-γ,γ-dimethylallyl ether, piperitol-γ,γ-dimethylallyl ether, pluviatol-γ,γ-dimethylallyl ether	[508]
					Bark: chelerythrine	[509]

^a^ Non-native. ^b^ Commercial (dōTERRA) essential oil. ^c^ W. N. Setzer (unpublished).
